# Direct measurement of individual phonon lifetimes in the clathrate compound Ba_7.81_Ge_40.67_Au_5.33_

**DOI:** 10.1038/s41467-017-00584-7

**Published:** 2017-09-08

**Authors:** Pierre-François Lory, Stéphane Pailhès, Valentina M. Giordano, Holger Euchner, Hong Duong Nguyen, Reiner Ramlau, Horst Borrmann, Marcus Schmidt, Michael Baitinger, Matthias Ikeda, Petr Tomeš, Marek Mihalkovič, Céline Allio, Mark Robert Johnson, Helmut Schober, Yvan Sidis, Frédéric Bourdarot, Louis Pierre Regnault, Jacques Ollivier, Silke Paschen, Yuri Grin, Marc de Boissieu

**Affiliations:** 1 Institut Laue-Langevin, Grenoble, F-38000 France; 2 University Grenoble Alpes, CNRS, Grenoble-INP, SIMaP, F-38000 Grenoble, France; 3 University Lyon, University Claude Bernard Lyon 1, CNRS, Institute of Light and Matter, F-69622 Villeurbanne, France; 4 Institute of Materials Science and Technology, Vienna University of Technology, 1040 Vienna, Austria; 5 Max-Planck-Institut für Chemische Physik fester Stoffe, 01187 Dresden, Germany; 6 Institute of Solid State Physics, Vienna University of Technology, 1040 Vienna, Austria; 7 Institute of Physics, Slovak Academy of Sciences, 84511 Bratislava, Slovakia; 8 Physikalisches Institut, Goethe-University, 60438 Frankfurt, Germany; 9 University Grenoble Alpes, UFR PhITEM, F-38000 Grenoble, France; 10 Laboratoire Léon Brillouin, CNRS, CEA, UMR-12, 91191 Gif sur Yvette, France; 11 University Grenoble Alpes, CEA, INAC, F-38000 Grenoble, France

## Abstract

Engineering lattice thermal conductivity requires to control the heat carried by atomic vibration waves, the phonons. The key parameter for quantifying it is the phonon lifetime, limiting the travelling distance, whose determination is however at the limits of instrumental capabilities. Here, we show the achievement of a direct quantitative measurement of phonon lifetimes in a single crystal of the clathrate Ba_7.81_Ge_40.67_Au_5.33_, renowned for its puzzling ‘glass-like’ thermal conductivity. Surprisingly, thermal transport is dominated by acoustic phonons with long lifetimes, travelling over distances of 10 to 100 nm as their wave-vector goes from 0.3 to 0.1 Å^−1^. Considering only low-energy acoustic phonons, and their observed lifetime, leads to a calculated thermal conductivity very close to the experimental one. Our results challenge the current picture of thermal transport in clathrates, underlining the inability of state-of-the-art simulations to reproduce the experimental data, thus representing a crucial experimental input for theoretical developments.

## Abstract (web summary)

Phonon lifetime is a fundamental parameter of thermal transport however its determination is challenging. Using inelastic neutron scattering and the neutron resonant spin-echo technique, Lory et al. determine the acoustic phonon lifetime in a single crystal of clathrate Ba7.81Ge40.67Au5.33.

## Introduction

A central issue in the development of modern nano- and microtechnology is the engineering of thermal conductivity of semiconducting materials. Thermal energy here is mainly carried by atomic vibrations, described by quasiparticles called phonons. During its lifetime, a phonon transports a quantum of heat, given by the product of its energy and its velocity. The knowledge of individual phonon properties is thus fundamental for applications such as waste heat recovery through thermoelectric (TE) conversion, where one of the main challenges is to identify semiconducting materials with low thermal conductivity^1–3^. As a rule of thumb, low thermal conductivity can be achieved by lowering phonon lifetimes, a strategy which has led to an intensive research activity in the ‘phonon engineering’ of TE materials. While phonon energies and velocities can be relatively easily measured and calculated, to access phonon lifetimes is extremely challenging both experimentally, because of the limited instrumental resolution of state of the art experimental techniques, and theoretically, as it requires the determination of anharmonicity and a detailed modeling of disorder. Recently, considerable progress has been made in ab initio computational methods, mainly on semiconductors with relatively simple structures^4–12^. In these systems the lifetime reduction due to three-phonon Umklapp processes has been calculated and successfully compared to experimental values of the thermal conductivity. As an example, phonons mostly contributing to the thermal conductivity were reported to have at room temperature mean free paths (MFP) in the 7–70 nm range in Bi_2_Te_3_ (5 atoms per unit cell) and 2–10 nm in PbTe, with a *1/T* temperature dependence in the high temperature limit. The effect of mass disorder has been calculated only in the case of simple element alloys^11, 13–15^, such as Si_0.5_Ge_0.5_, where the relevant mean free path was found to be 0.1–5 μm.

Face to these recent computational advances, it is necessary to validate the theoretical predictions by actual measurements of the lifetimes of individual phonon states. The phonon lifetime is related to the intrinsic inelastic scattering signal width (full width at half maximum FWHM) by the relation *τ* ^−1^ = *π*.Γ_FWHM_, where Γ_FWHM_ is the signal FWHM expressed in THz^16^. A typical inelastic neutron scattering (INS) experiment allows measuring lifetimes up to *τ* ~ 5 ps (corresponding to a phonon energy full-width at half maximum Γ_FWHM_ ~ 250 µeV), which give a MFP of the order of 10 nm for a phonon group velocity of 2000 m.s^−1^. As a result, effective phonon lifetimes have been accurately measured only in a few cases where they are already very short, such as in PbTe (Γ_FWHM_ ~ 400 μeV, *τ* ~ 3 ps for *E* ~ 1.5 meV)^17–19^ and Na_0.8_Co_2_ (Γ_FWHM_ ~ 625 μeV, *τ* ~ 2 ps)^20^ or in nanostructured materials with nanometric grains such as AgSbTe (Γ_FWHM_ ~ 500 μeV, *τ* ~ 2.6 ps for *E* ~ 1.5 meV)^19, 21^. The neutron resonant spin-echo technique (NRSE) allows for accessing much longer lifetimes, but up to now it has been applied only to simple systems such as Pb and Nb, where lifetimes in the range 25–90 ps (Γ_FWHM_ = 15−5 μeV) have been reported^22^.

**Fig. 1:**
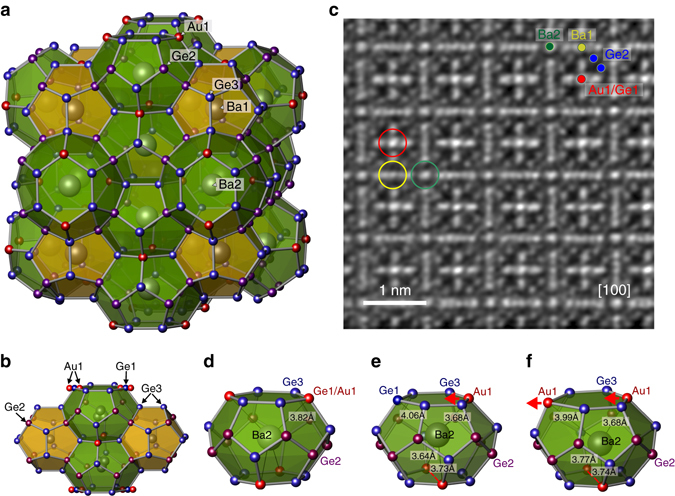
Crystal structure and local atomic arrangements of Ba_7.81_Ge_40.67_Au_5.33_. **a** Ordered clathrate-I model with the composition Ba_8_Ge_40_Au_6_ showing the three-dimensional Ge/Au framework with 20-vertices polyhedra (*orange*) and 24-vertices polyhedra (*green*), occupied by Ba1 (Wyckoff site *2a*) and Ba2 (*6d*) atoms, respectively. Au atoms on the *6c* site are shown in *red*. **b** Atomic arrangements around Ba2 and Ge1/Au1 positions; *red arrows* mark the shift of the Au1 atoms with respect to the *6c* site. **c** Atomic-resolution Scanning Transmission Electron Spectroscopy (STEM) image along the [100] direction reveals strong displacement of atoms from the ideal positions in the regions of Ba2 (*green*) and Au1/Ge1 (*red*) positions in comparison with the Ba1 ones (*yellow*). Additional deviations from the translational symmetry can be recognized in several regions of the image. **d** Ideal ordered arrangement in the Ba2 polyhedron with Ba2 in *6d* site and equivalent Ba2-Ge1-Au1 distances for a hypothetical undistorted model. **e** A possible atomic arrangement in the Ba2 polyhedron with three Au atoms at Au1 site, Ba2 displaced from the *6d* site and smaller Ba2-Au1 distances due to the Ba-Au interactions; **f** Atomic arrangement with 3 Au atoms as obtained by using positions from ab initio calculation on a 2×2×2 supercell and the experimental lattice parameter. The distances compare well with experimental data in **e**

Low thermal conductivities have also been observed in structurally complex crystals with a large number of atoms in the unit cell (> 50 atoms), such as guest–host structures (clathrates and skutterudites), where however phonon lifetimes result beyond the standard resolution limit. These materials, known as cage compounds, are composed of covalently bonded polyhedra filled by guest atoms (see Fig. 1a for clathrates) and are characterized by a low thermal conductivity of typically *κ* about 1–2 W m^−1^ K^−1^ at room temperature^23^, which is assumed to be related to the presence of low-energy, non-dispersive optical phonons^24–29^. Formerly associated with isolated vibrations of the guest atoms^26, 30^, more recent investigations indicate that these optic modes hybridize with the host matrix, leading to an energy cutoff for the propagative acoustic phonons, and their localisation above it^27, 29^. Moreover, in some clathrates the typical Umklapp peak in the *κ*(*T*) dependence is smoothed out and replaced by a weakly temperature dependent ‘plateau’^23, 31^, that has frequently been referred to as ‘glass-like’. In glasses, this is due to very short phonon mean free paths, which have been supposed to exist also in clathrates, possibly caused by two-phonon processes like resonant scattering on guest atoms^32, 33–37^, or three-phonon processes due to anharmonicity. Indeed, ab initio calculations in clathrates and other complex material^37–40^ indicate that the many low-lying optic modes enhance the occurrence of three-phonon processes, reducing the acoustic phonons lifetime. However, to date, no experimental evidence of a marked reduction in phonon lifetimes has been found via inelastic neutron/X-ray scattering techniques, mainly because of the limited energy resolution achieved in those experiments^24, 26, 27, 29^.

Here, we tackle this problem, by successfully achieving the hard task of measuring the longest ever reported lifetimes for acoustic phonons in a large single crystal of the clathrate Ba_7.81_Ge_40.67_Au_5.33_ (about 54 atoms in the unit cell) whose measured lattice thermal conductivity at 300 K is of 1.1 W m^−1^ K^−1^ (see Methods and Supplementary Note 3). This result has been possible thanks to the remarkable crystalline quality (bulk mosaicity of 0.01°, see Methods and Supplementary Note 1) achieved, allowing for unprecedented high-resolution measurements of the acoustic phonon lifetime by means of INS and NRSE. These measurements are supported by ab initio density functional theory (DFT) simulations, and allow us to experimentally confirmed of the long-established thermal transport modeling in complex systems.

## Results

### Structural characterization of Ba_7.81_Ge_40.67_Au_5.33_

The importance of achieving a detailed understanding of the atomic structure, including disorder, has been recently highlighted for AgSbTe^21^, where deviations from the ideal, periodic model significantly affect thermal properties.

Ba_8_Ge_46-*x*_Au_*x*_ is a clathrate of type I (space group $Pm\overline 3 n$), made of two kinds of face-sharing Ba-filled cages: a dodecahedral one (20 vertices) and a larger tetrakaidecahedral one (24 vertices; Fig. 1a). At the hypothetical composition Ba_8_Ge_40_Au_6_ and in a fully ordered model, 4 gold atoms substitute Ge in this latter cage (*6c* site), in a distorted tetrahedral arrangement around the central Ba atom (Fig. 1b), however significant deviations from this ideal ordered model have been previously reported^41^. We have here performed a detailed X-ray diffraction investigation, which confirms these findings, revealing that the composition of the unit cell is Ba_7.81_Ge_40.67_Au_5.33_ (Supplementary Note 2) and shows that the Ba2 atoms in the large cages are off-centred and do not occupy the high symmetry position (*6d*) any more. They are at an average distance of 0.26 Å from the ideal one, the site being occupied at 96.8%, with a typical inter-defect (vacancy) distance of about 4 nm. Moreover, as a consequence of the covalent Ba–Au bonding^41^, Au atoms substitute Ge atoms only partially (about 88.4%) and are off-centred by 0.08 Å out of the high symmetry (*6c*) position, the corresponding characteristic inter-defect distance being of approximatively 1.5 nm.

High-end transmission-electron microscopy and STEM images on the same crystal (see Methods) confirmed clearly those results, with Ba2 and Au/Ge1 atoms displaced from their ideal positions, resulting in deviations from the translational symmetry with a wide spectrum of characteristic distances as seen Fig. 1c.

The disorder has been studied by ab initio DFT simulations at 0 K and by DFT molecular dynamics at 300 K (Supplementary Note 2). A stable atomic arrangement was obtained using a 2×2×2 supercell model with chemical composition close to the experimental one and randomly distributed Ge and Au atoms. About half of the Ba2 atoms in the large cages are found to be surrounded only by three gold atoms, with the Ba2 shifting by 0.27 Å towards the Au-triangle, in good agreement with the experimentally determined structure (Fig. 1d–f). A good agreement was also obtained between the experimental structure and the time averaged structure simulated by DFT molecular dynamics.

### Measurements of the phonon spectra in Ba_7.81_Ge_40.67_Au_5.33_

**Fig. 2:**
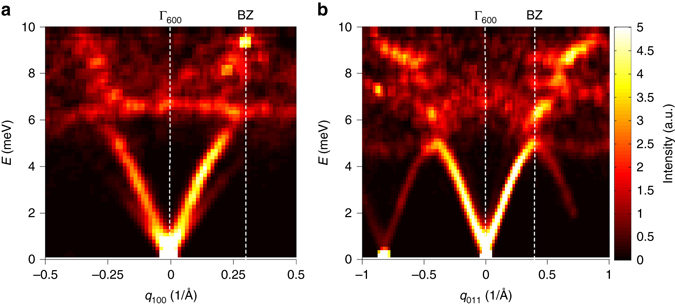
Experimental surface mapping of the longitudinal acoustic (LA) and transverse acoustic (TA) phonon dispersions in Ba_7.81_Ge_40.67_Au_5.33_ measured at 300 K. Two-dimensional inelastic neutron scattering intensity distribution as measured on the neutron time-of-flight spectrometer (IN5@ILL) at room temperature around the strong zone center ${\Gamma _{600}}$ (*Q* = 3.6 Å^−1^). **a** LA phonons measured along the [100] direction. Well-defined propagating excitations are only observed for LA modes with an energy lower than 6 meV. **b** TA phonons propagating along [011] and polarized along [100]. Well-defined propagating excitation are only observed for TA modes with an energy lower than 5 meV

**Fig. 3:**
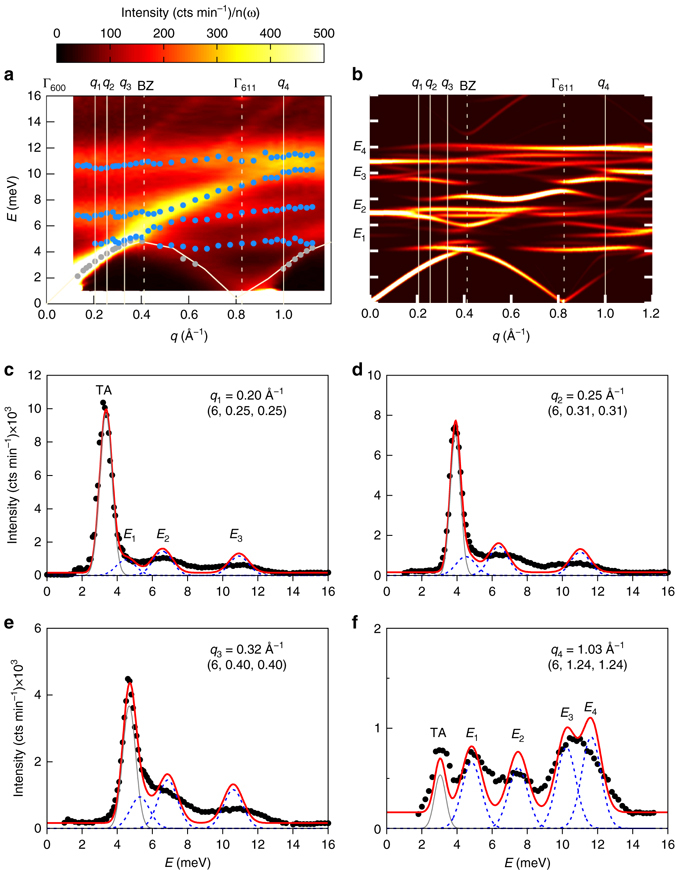
Transverse acoustic (TA) phonon spectra in Ba_7.81_Ge_40.67_Au_5.33_: experiment vs. simulation at 300 K. **a** Two dimensional neutron intensity distribution as experimentally measured on a neutron triple axis spectrometer (2T@LLB), along the reciprocal direction (6, *h*, *h*) from the Brillouin zone centre Γ${{\rm{\Gamma }}_{600}}$ (*Q* = 3.6 Å^−1^) showing the ${\rm{TA}}_{011}^{100}$ dispersion and its hybridization with low-lying optic Ba and Au modes (*red* is higher intensity). *Grey* and *blue full circles* stand for acoustic and optical excitations respectively as extracted from individual constant q-scans. The *solid white line* is the ${\rm{TA}}_{011}^{100}$ dispersion relation fitted from the individual constant q-scans as reported in **c**–**f**). **b** Two-dimensional neutron intensity distribution obtained by harmonic simulation in the density functional theory. A different scaling factor has been applied for the acoustic (1.4) and optical (1.2) contributions (see text). Experimental points are shown as *grey* (acoustic) and *blue* (optic) *full circles*, respectively. **c**–**f** example of four constant *q*-scans, *q*_1_ to *q*_4_ as indicated by the white vertical lines in the mapping (**a**, **b**). Experimental data (*full black circle*) are compared with ab initio phonon calculations after convolution with the instrumental resolution (*red line*). Calculated, individual phonon modes are reported in *light grey* (acoustic) and *blue dotted line* (optic). The energy scan at Γ${{\rm{\Gamma }}_{611}}$ (**f**), for which the acoustic intensity is low, makes possible a direct observation of the low-lying optical intensities analysed using four Gaussian profiles centered at energies *E*_1_, *E*_2_, *E*_3_ and *E*_4_

Lattice dynamics investigations were carried out by INS, on the same single crystal Ba_7.81_Ge_40.67_Au_5.33_, and by ab initio calculations using an ideally ordered cubic model, Ba_8_Ge_40_Au_6_ (see Methods and Supplementary Notes 4 and 5). Longitudinal (LA) and transverse (TA) acoustic phonons were measured around the strong Bragg peak (6,0,0). Figure 2 reports the *S*(**Q**, *E*) intensity distribution measured at 300 K around the zone centre Γ_600_ for LA_011_ modes propagating along the (0,1,1) direction (Fig. 2a) and for ${\rm{TA}}_{011}^{100}$ modes, propagating along (0,1,1) and polarized along (1,0,0) (Fig. 2b) on a neutron Time Of Flight (TOF) spectrometer. Similarly to other clathrates^27, 29^, for both transverse and longitudinal excitations, there are well-defined propagative acoustic excitations only in a limited range below a low-lying optical excitation. Figure 3 displays raw data (mapping of the phonon dispersions and constant Q-scans) measured on a neutron Triple Axis Spectrometer (TAS) at 300 K over a larger energy range, together with a comparison with simulated spectra convoluted with the experimental resolution for ${\rm{TA}}_{011}^{100}$ modes covering several Brillouin zones (all constant *Q*-scans are shown in the Supplementary Note 17). Similarly to other clathrates, the phonon spectra in Ba_7.81_Ge_40.67_Au_5.33_ can be separated into two energy regimes on either side of the lowest lying optic mode, labeled *E*_1_. With an energy of 4.5 meV, this mode is lower than the one in Ba_8_Ge_42.1_Ni_3.5_ (measured at 5.5 meV^29^, or in Ba_8_Si_46_ (observed at 7 meV^27^), confirming ab initio predictions. It thus suppresses more acoustic states, in agreement with a lattice thermal conductivity at room temperature lower than in lighter transition metal (TM) substituted Ge clathrates without Ba-TM bonding (see Supplementary Note 5 and refs. ^41–44^). Below *E*_1_, well-defined acoustic modes are found which continuously disperse up to *E*_1_, keeping a pure acoustic character only for energies lower than 3 meV, similarly to other clathrates^26, 27, 29^. Approaching *E*_1_, the acoustic branches transfer their spectral weight to the optic modes, and bend over, their slope approaching zero at the Brillouin zone boundary (Figs. 2b and 3a,b). Above 4.3 meV the phonon spectrum is dominated by optic modes, and can be modeled with four Gaussian distributions labeled *E*_1,2,3,4_, describing the superposition of several weakly dispersive optical phonon branches and located at energies of 5, 7.5, 10 and 12 meV respectively. As observed in other clathrates, depending on the dynamical range of the color mapping, the spectral weight transfer can be clear, as in Fig. 2 here, or can be hidden by an apparent going-on dispersion shared between the acoustic and the optic modes, as seen in Fig. 3a.

Our ab initio DFT simulation well reproduces the acoustic and optic phonon dispersions and the experimental intensity distribution (Fig. 3), a much constraining test. However, in order to achieve a good agreement we had to apply an energy scaling factor equal to 1.4 for the acoustic part (*E* < 6 meV) and 1.2 for modes at higher energies, similar to what was used for Ba_8_Ge_42.1_Ni_3.5_^29^. This might be ascribed to an underestimation of the Ba/Au-Ge interactions within the DFT approach.

Ab initio DFT simulations allow us to compute the contribution of the different sites to the signal: we can thus assign the four *E*_1,2,3,4_ optical excitations respectively to Ba2-, Au-, Ge-, Ba1/Ge- dominated vibrations, as shown by the partial vibrational density of states (Supplementary Note 5 and Supplementary Fig. 11). One should also notice that including the experimentally observed disorder into the simulation is expected to broaden the simulated optical excitations^24^.

### Measurements of the phonon lifetimes in Ba_7.81_Ge_40.67_Au_5.33_

**Fig. 4:**
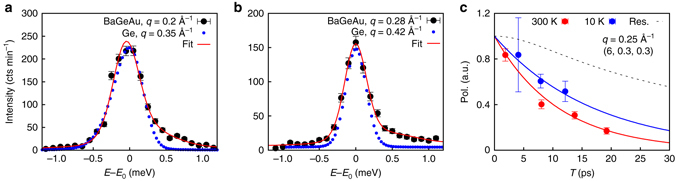
Experimental measurements of transverse acoustic (TA) phonon lifetimes in Ba_7.81_Ge_40.67_Au_5.33_ (BaGeAu). **a**, **b** Comparison between the ${\rm{TA}}_{011}^{100}$ phonons in BaGeAu clathrate (*black points*) and in pure Ge (*blue dotted line*) measured with constant *q*-scans, at *q* = 0.2 Å^−1^ and *q* = 0.28 Å^−1^ where their local group velocities are identical. Measurements were performed on the triple axis spectrometer (TAS) 2T@LLB with an effective resolution of 200 μeV. The Ge data represent directly the effective TAS resolution. Data are reported as a function of *E*–*E*_0_, with *E*_0_ the acoustic phonon mode energy. In **a**, *E*_0_ = 2.97 meV (BaGeAu) and *E*_0_ = 5.72 meV (Ge); in **b** *E*_0_ = 4 meV (BaGeAu) and *E*_0_ = 6.75 meV (Ge). **c** Evolution of the neutron polarization as a function of *τ*_NRSE_ for the ${\rm{TA}}_{011}^{100}$ phonon (*q* = 0.25 Å^−1^, *E* = 3.8 meV) measured with the Neutron Resonant Spin-Echo (NRSE) technic at 300 K (*full red circle*) and at 10 K (*full blue circle*) measured on the spectrometer IN22-ZETA@ILL. Data are corrected for the experimental resolution shown as a *dotted line*. The *full blue* and *red lines* are exponential fits to the data from which phonon lifetimes equal to 33(10) ps and 15(1) ps at 10 and 300 K are extracted. Vertical error bars in **a**–**c** are s.d. arising from counting statistics

Quantitative measurements of acoustic phonon lifetimes are extremely challenging. The key point is the central role of the instrumental resolution function, which depends both on the wave vector **q** and the energy *E* so that, for dispersive excitations, the observed peak width depends on the local curvature of the phonon dispersion *E*(**q**) and thus on the local group velocity (see Supplementary Note 4, Supplementary Fig. 5 and refs.^27, 29^). Benefiting from the large crystal volume, our first approach was to use a low-flux neutron TAS setup in order to achieve a high instrumental resolution equal to 200 μeV (FWHM) for the ${\rm{TA}}_{011}^{100}$ modes, giving access to intrinsic broadenings larger than 50 μeV. Data collected at 300 K are shown in Fig. 4a, b (more constant *q*-scans are shown in the Supplementary Figs. 7, 17 *)*. Although for phonons with *q* < 0.22 Å^−1^ (*E* < 3.3 meV), the peak width is resolution limited, a sizeable broadening of about 100 μeV is observed for wave vectors between 0.25 and 0.32 Å^−1^. At higher wave vectors, acoustic and optic peaks overlap, increasing the fit uncertainty on the phonon linewidths determination. To validate this result, we directly compare the intensity profile of the clathrate acoustic phonons with those measured under exactly the same conditions on a pure Germanium crystal, as shown Fig. 4a, b. Whereas both excitations are almost identical for the phonons measured at *q* = 0.2 Å^−1^ (Fig. 4a) a sizeable broadening is observed at 0.28 Å^−1^ in the clathrate (Fig. 4b), confirming that the measured widths for *q* > 0.22 Å^−1^ (*E* > 3.3 meV) are intrinsic.

To overcome the limited resolution of TAS experiments and measure lifetimes for phonons below 4 meV, the NRSE technique was used, in a configuration giving access to phonon lifetimes up to 66 ps, corresponding to an energy resolution of 20 µeV^22^ (see Methods). Figure 4c shows a typical NRSE result from which the phonon lifetimes τ are extracted. In Table 1, we report the measured values for the ${\rm{TA}}_{011}^{100}$ branch at 300 K, at four phonon energies between 2 and 4.4 meV.

**Table 1:** Summary of the experimental ${\bf{TA}}_{{\bf{011}}}^{{\bf{100}}}$ individual phonons properties Ba_7.81_Ge_40.67_Au_5.33_

*T* (K)	**q** (0, ξ, ξ) (r.l.u.)	*q* (Å^−1^)	*E* (meV)	Γ_FWHM_ (μeV)	*τ* (ps)	*V*_G_ (m s^−1^)	*l* (nm)
300	0.143	0.11	2	25 (2)	53 (3)	2260	120
300	0.24	0.19	3.2	82 (7)	16 (1)	1950	31
300	0.30	0.25	3.8	87 (7)	15 (1)	1690	25
300	0.36	0.29	4.4	96 (12)	14 (1)	1530	21
10	0.143	0.11	2	24 (10)	55 (17)	2260	124
10	0.30	0.24	3.8	40 (13)	33 (11)	1690	56

The columns report temperature *T* (K), the phonon wavevector *q* in r.l.u. and in Å^−1^, the phonon energy *E* in meV, the phonon FWHM width Γ_FWHM_ (μeV), the phonon lifetime *τ* in ps, the phonon group velocity in m.s^−1^ and the phonon mean free path *l* in nm. The relationship between the phonon width in energy Γ_FWHM_ (μeV) and the lifetime *τ* (ps) is $\tau {\left({{\rm{ps}}} \right)^{ - 1}} = \frac{{{\Gamma _{{\rm{FWHM}}}}}}{{2\hbar }} = {0.7592.10^{ - 3}}.{\Gamma _{{\rm{FWHM}}}}\left({{\rm{\mu e}}V} \right)$ ^21, 27^

**Fig. 5:**
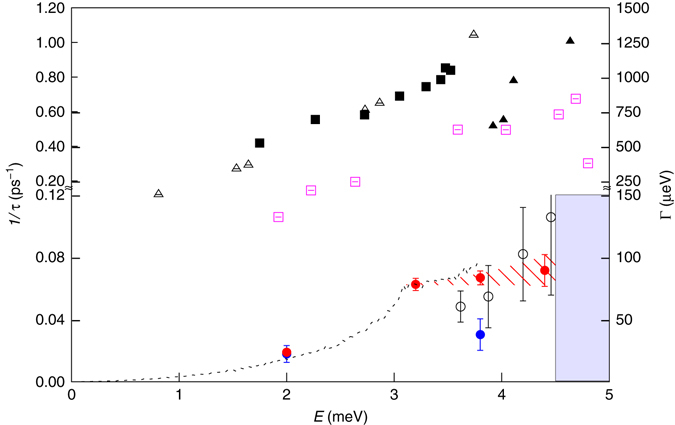
Energy and temperature dependences of transverse acoustic (TA) phonon lifetimes in Ba_7.81_Ge_40.67_Au_5.33_. ${\rm{TA}}_{011}^{100}$ inverse phonon lifetimes (*left*) and energy Full Width at Half Maximum (FWHM, *right*) as determined from measurements on the neutron triple axis spectrometer (TAS, *black circles*) and the neutron resonant spin-echo (NRSE) spectrometer as a function of energy at 300 K (*full red circles*) and at 10 K (*full blue circles*). Above 4.5 meV the extraction of a single acoustic phonon mode lifetime is no longer possible due to the proximity of a low lying optical excitation (*blue shaded area*). Vertical error bars are standard deviation obtained by error propagation during the fitting procedure. The dotted line shows the rescaled calculated phonon density of state (p-DOS(*ω*), in meV^−1^; see Supplementary Note 5) which is truncated at *E* = 3.8 meV due to van Hove singularities. The calculated phonon lifetime from DFT (see text) for the ${\rm{TA}}_{011}^{100}$ mode is shown as (*empty pink squares*). Previous experimental determination of acoustic phonon lifetimes in the same energy range are reported for PbTe (*full blac﻿k squares*, Delaire et al.^17^), AgSbTe_2_ (*empty triangles*, Ma et al.^19, 21^) and amorphous Silica (*full black triangle﻿s*, Baldi et al.^48^)

Figure 5 summarizes our results, showing the energy dependence of our TAS and NRSE measurements of the inverse lifetime *1/τ* (and energy linewidth Γ_FWHM_) of acoustic phonons, and comparing them to published measurements in other complex crystalline and disordered materials in the same energy range. In our clathrate, 1/*τ* increases with energy up to 3.2 meV, where then it is roughly constant up to 4.4 meV. Interestingly, such a step-like behavior is also seen in the computed phonon density-of-states (p-DOS, black dotted line in Fig. 5). At the lowest energy value, *E* = 2 meV, a lifetime of 53(4) ps is found which, with the group velocity at this energy, corresponds to a phonon mean free path of 120 nm. At *E* = 3.2 meV, 3.8 and 4.4 meV, the phonon lifetimes are of the order 15 ps, and the phonon mean free paths are 31, 25 and 21 nm, respectively, i.e. more than 10 times larger than the unit cell (*a* = 1.08 nm). The measured lifetimes are much smaller than in pure Ge where, from the measured *τ* about 66 ps at 9.8 meV^45^, we can estimate *τ* about 660 ps at 3 meV. Our results are also compared in Fig. 5 to previously obtained data in other thermoelectrics such as PbTe^17–19^ or AgSbTe_2_^19, 42^ and amorphous Silica^46–48^ . For the clathrate Ba_7.81_Ge_40.67_Au_5.33_ and in the energy range 2–4 meV we find much larger lifetimes than in all these compounds.

It is also interesting to compare the acoustic mean free path here found with the ones of the optical excitations *E*_2_, *E*_3_ and *E*_4_, whose experimental lifetime is of the order of 1 ps, assuming that a single excitation contribute to each of these modes (Supplementary Note 4). Optical excitations are almost dispersionless or weakly dispersing (Fig. 3), so that their mean free path is extremely short. For the excitation *E*_2_ its maximum value is equal to 1.5 nm, i.e., more than one order of magnitude lower than for the 4.4 meV acoustic excitations.

**Fig. 6:**
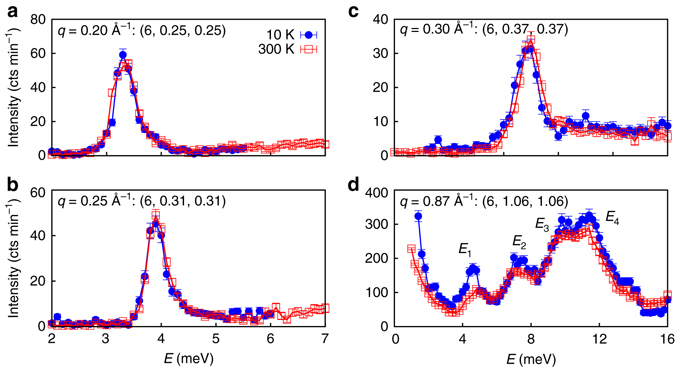
Transverse acoustic (TA) phonons profiles measured at 10 and 300 K in Ba_7.81_Ge_40.67_Au_5.33_. **a**–**c** Comparison between constant *q*-scans of the ${\rm{TA}}_{011}^{100}$ branch measured at 10 K (*full blue circles*) and 300 K (*red squares*) on the triple axis spectrometer (called 2T@LLB) with an effective resolution of 200 μeV. **d** Similar comparison between scans with a larger resolution of 1.2 meV measured at the wave vector (6, 1.06, 1.06) close to the centre of the BZ where the four optic phonon modes, labeled *E*_1,2,3,4_ in the text, are clearly visible (**a**, **b**). In **a**–**d**, the intensities are corrected for the Bose population factor. *Vertical error bars* are s.d. from counting statistics

Finally, we have investigated the temperature dependence of the phonon lifetime, by repeating the neutron TAS and NRSE measurements at 10 K at the same conditions during the same experiments. Figure 6 compares the neutron TAS intensity profiles measured at 300 K and 10 K for those phonons for which an intrinsic linewidth was found. With the TAS, the sensitivity being limited at 50 μeV (Supplementary Note 4 and Supplementary Fig. 5), we cannot resolve any temperature dependence of the phonon linewidth, but we demonstrate the absence of a strong shift of the phonon energies, confirming previous results at 300 K in clathrates^27, 29^, which show that the guest phonon modes are coherently coupled with the host lattice without anharmonicity. NRSE reveals a temperature effect of the phonon lifetime at *E* = 3.8 meV, which decreases from 33(10)   ps at 10 K to 15(1) ps at 300 K (Fig. 5 and Table 1), while no temperature effect is observed at 2 meV, whose value at 300 K is already at the limit of detection.

### A simple phenomelogical model for the thermal conductivity in complex materials

Using these experimental results we can calculate the lattice thermal conductivity *κ*_L_, which, in the framework of the relaxation-time approximation of the Boltzmann equation, is given by:

1 $${\kappa _{\rm{L}}} = \frac{1}{3}\mathop {\int }\limits_0^{{\omega _{{\rm{max}}}}} {C_{\rm{v}}}\left(\omega \right){v^2}\left(\omega \right)\tau \left(\omega \right)\rho \left(\omega \right){\rm{d}}\omega $$

where *C*_v_(*ω*), *v*(*ω*) = ∂*ω*/∂*q*, *τ*(*ω*) and *ρ*(*ω*) are the phonon specific heat per unit volume, velocity, lifetime and phonon density of states respectively.

Instead of considering the 3 × 54 phonon modes we assume, as was proposed for complex dielectrics^49–51^, that only the three acoustic branches contribute to *κ*_L_. Indeed, above the acoustic cut-off frequency, the measured optical excitations are both weakly dispersive and rather broad with lifetimes much shorter than the one of acoustic modes, thus leading to minor contributions to the lattice thermal conductivity and to major contributions to the heat capacity. The cutoff frequency for a given acoustic branch, *ω*_max_, is such that $\mathop {\int }\limits_0^{{\omega _{{\rm{max}}}}} \rho \left(\omega \right){\rm{d}}\omega = 1$ and is equal to 4.7 meV for TA modes, in agreement with the experimental dispersion relation (Figs. 2, 3).

We then assume a phenomenological energy dependence of the acoustic phonon lifetimes: for *ħ*ω < 3.2 meV, it mimics the one of the p-DOS(*ω*) (*black dotted line* in Fig. 5), *τ*(*ω*) ∝ p−DOS(*ω*), while for 3.2 meV ≤ *ħ*ω < *ħ*ω_max_, the lifetime is fixed at 16 ps. The acoustic phonon dispersions are assumed isotropic, following experimental results here and in other clathrates^29^. With these assumptions, Eq. 1 yields at room temperature *κ*_L_ = 1.2 W m^−1^ K^−1^ in very good agreement with the experimental value (1.1 W m^−1^ K^−1^).

This confirms previous studies showing that in complex dielectric crystals *κ*_L_ is well approximated by considering only the acoustic phonons, even though the optic modes mainly contribute to the heat capacity, here more than 98% (the Dulong-Petit limit gives $C_{{\rm{DP}}}^{{\rm{op}}}$ = 259.47 mJ g^−1^ K^−1^ while $C_{{\rm{DP}}}^{{\rm{ac}}}$ = 4.91 mJ g^−1^ K^−1^), and provide scattering channels for acoustic phonons^3, 21, 52, 53^. This approximation works especially well in guest–host materials where atomic masses and chemical bonds differ substantially. We can thus understand thermal transport in clathrates as essentially due to acoustic modes, propagating in a small energy range, limited by the lowest-lying optic mode, and on distances of a few tens of nanometers. In agreement with recent studies in clathrates, guest modes act as a ‘low band pass’ filter for acoustic phonons^27, 29^. This model also works in intermetallic quasicrystals and their periodic approximants, where an almost temperature independent thermal conductivity in the W m^−1^ K^−1^ range is observed. Here, the energy cutoff limiting the acoustic regime is 5–7 meV^50, 54, 55^ resulting in mean free paths of 20–30 nm for phonon wave-vectors below 0.3 Å^−1^, in agreement with recent estimations^53^. Note that a valid first-order approximation of the thermal conductivity can be obtained in the classical kinetic gas limit of Eq. 1, i.e., the Debye model, considering only the three acoustic phonons and a constant phonon lifetime (Supplementary Note 6). The energy-dependent acoustic contribution can be estimated looking at the accumulated thermal conductivity ${\kappa _{\rm{L}}}\left(\omega \right) = \frac{1}{3}\mathop {\int }\limits_0^\omega {C_{\rm{v}}}\left(\omega \right){v^2}\left(\omega \right)\tau \left(\omega \right)\rho \left(\omega \right){\rm{d}}\omega$. About 50% of the thermal conductivity is carried by phonon in the range 0–2 meV, or the range 2–4.5 meV (Supplementary Note 6 and Supplementary Fig. 15). The acoustic phonons lying in the linear part of the dispersion, with *E* < 2 meV and the largest velocities, have a larger relative weight at lower temperature. These phonons, with energies lower than the energy difference between the lowest optical modes, $E_2^{{\rm{optic}}} - E_1^{{\rm{optic}}}$~2 meV, are less affected by the three-phonon scattering processes involving these optical modes (acoustic + optic(1) <–> optic(2)), as determined by the energy and momentum conservation laws^3^.

### Theoretical simulations of the three-phonon anharmonic scattering processes in the clathrate BaGeAu

Identifying what are the underlying mechanisms responsible for the observed phonon lifetimes requires an analysis of both their energy and temperature dependence. There are four possible phonon scattering processes that can be considered: three-phonon anharmonic processes, scattering on defects, scattering on electrons and scattering on domain boundaries^56^. On one hand our experimental results do not allow to uniquely identify the different processes, on the other hand very little has been done on simulations of structurally complex and disordered materials as explained in the introduction. For instance, models for disorder are only available for the so-called mass defects in simple structures as Ge^11, 13, 14^ and DFT electron–phonon calculations have only been achieved recently for Si^57^. Simple anharmonic or disordered models can neither account for the observed relatively small temperature dependence nor for the rather unexpected frequency dependence of the phonon lifetimes.

As a first attempt to computationally tackle these issues, we have however calculated the three-phonon scattering rates and corresponding phonon lifetime contributions using DFT calculations and the method recently proposed and applied by Tadano et al.^40^ on similar clathrates. We performed the simulation on a single unit cell with the ideal composition Ba_8_Ga_40_Au_6_ without any disorder, i.e., Ba atoms are located at the cage centre and there is no Au/Ge disorder (Supplementary Note 5). Indeed, current computation limitations do not allow for calculations on a 2 × 2 × 2 supercell that would be required for describing the observed disorder.

**Fig. 7:**
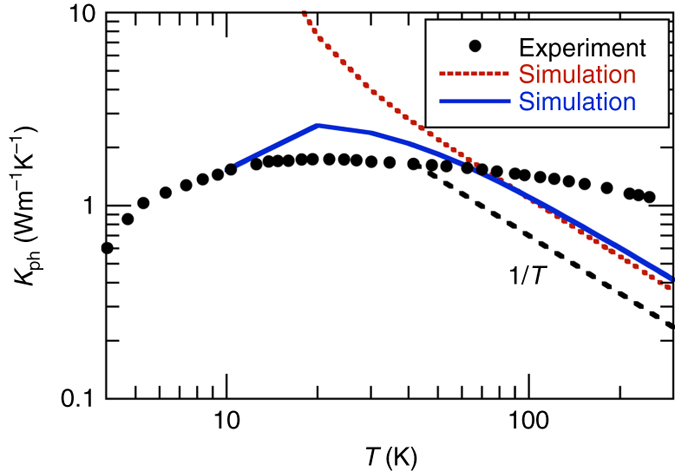
Lattice thermal conductivity in Ba_7.81_Ge_40.67_Au_5.33_. Comparison between the experiment (*full circles*) and the anharmonic three phonon simulation (*red dotted line*) carried out using the experimental lattice parameter. The *blue solid line* includes boundary effect with a size of 500 nm. The *dashed black line* indicates the 1/*T* decay. Other scattering mechanisms (defects, electron-phonon) needs to be included at temperature lower than 50 K

Figure 7 reports the temperature dependence of the experimental and simulated phonon lattice thermal conductivity, while phonon lifetimes are presented in the Supplementary Note 5. In order to roughly reproduce the low *T* part of the observed thermal conductivity (*T* < 50 K), we have introduced boundary effect, with grain size of 500 nm as shown by the blue line in Fig. 7. This size is likely too small, and other mechanisms (electron–phonon and defects) should be included to explain the low *T* behavior. However, this is beyond the scope of the present work, where we concentrate on the high temperature dependence, for *T* larger than 50 K.

Our simulations are qualitatively and quantitatively very similar to the results obtained by Tadano et al. for Ba_8_Ga_16_Ge_30_ in ref. ^40^ and display a number of discrepancies with our experimental results. First of all, the calculated room temperature lifetimes and lattice thermal conductivities are smaller than the experimental values by a factor 3 to 5 as exemplified in Figs. 5, 7.

A similar discrepancy is actually also observed in the simulation of Tadano et al. for Ba_8_Ga_16_Ge_30_ in ref. ^40^ if temperatures larger than 100 K are considered (at 300 K the experimental value is equal to 1.3 W m^−1^ K^−1^ in ref. ^58^ whereas the simulation in ref. ^40^ yields 0.4 W m^−1^ K^−1^). Note that a phonon lifetime equal to 2–3 ps, as predicted by the simulation at 4 meV, would be easily detected experimentally. Moreover, simulations carried out on simple models for the observed disorder further decrease the phonon lifetime and thermal conductivity, as expected (Supplementary Note 5, Supplementary Figs. 12 and 13), thus increasing the disagreement with the experiment.

The second point of disagreement is the temperature dependence of both phonon lifetimes and thermal conductivity, which in simulations scale both as 1/*T* for *T* larger than 40 K, simply reflecting Bose–Einstein statistics, while experimentally display a very weak dependence between 50 and 300 K (Fig. 7).

It is worth underlying, however, that despite all these points of disagreement, simulations rather well reproduce the experimentally observed energy dependence of the inverse lifetime, which in the long wavelength limit roughly scales with *ω* ^2^ (see Fig. 5 and Supplementary Note 5).

The origin of the discrepancy on the temperature dependence and the absolute values of phonon lifetimes and thermal conductivity needs thus to be sought elsewhere, and requires further measurements of both temperature and energy dependence of phonons lifetime, coupled to atomistic or molecular dynamics simulations taking into account all the present results, as well as a possibly temperature-dependent force field.

Indeed, the *1/T* decay in simulations is related to the Bose–Einstein phonon population factor, with the assumption that the force field does not change with temperature (see ref. ^9^). If this assumption is released, the temperature dependence of the simulated thermal conductivity could be different.

Concerning the absolute value of phonon lifetimes and thermal conductivity, the current phonon Umklapp simulations might underestimate them. Indeed is worth noticing that a slight change of the lattice parameter used in simulations can strongly affect them, as shown by Tadano et al.^10^ Going from the relaxed (1.10 nm) to the experimental lattice parameter (1.08 nm) leads to a factor 4 increase in the phonon thermal conductivity and average phonon lifetimes (see Supplementary Note 5 and Supplementary Figs. 13, 14). This is most likely associated with the underestimation of the phonon energies by the DFT calculation as already pointed out before.

Finally, the structural complexity and the large chemical disorder are not fully taken into account in the DFT simulation, for they likely are related to temperature independent two phonons scattering processes.

To summarize, our experimental results point to a profound disagreement with state of the art simulations and ask for future advanced calculations, better describing the results of our detailed structural analysis, including the large disorder, as well as a non-trivial temperature dependence of the 3-phonons scattering. In particular, molecular dynamics simulations as carried out by Tse et al. in clathrate hydrate^35, 59^ or in Ge clathrate using model Hamiltonian as in refs. ^28, 60^ allows to handle large system size^61^. We believe that the data provided here will give a robust starting point for such studies.

## Discussion

Our main result is the first accurate measurement of the acoustic phonon mean free path in a complex crystalline system, specifically Ba_7.81_Ge_40.67_Au_5.33_. To summarize our findings, at 300 K, the mean free path goes from 120 to 20 nm as the phonon wavevector and energy go from 0.1 to 0.3 Å^−1^ and 2 to 4.4 meV, respectively. As already pointed out, this is much larger than any other previously experimentally accessed phonon mean-free path in similar systems and in the energy range 2–4 meV.

It is thus interesting to compare these results with other thermoelectric systems for which DFT simulations give a good agreement with experiment, namely the PbTe^8^ system with two atoms per unit cell and the Bi_2_Te_3_^9^ one, with five atoms per unit cell. These two systems have room temperature thermal conductivities equal to 2 and 1.5 W m^−1^ K^−1^, respectively and in both cases simulations predict that about 80 % of the heat is carried by acoustic modes.

PbTe exhibits experimental acoustic phonon lifetimes much shorter than our case, despite a similar thermal conductivity at room temperature. It is important to note however that recent DFT calculations in PbTe^8^, in reasonable agreement with inelastic neutron scattering data^18^, report simulated acoustic phonon lifetimes roughly scaling with 1/*ω* ^*2*^, and with a value of the order of 10 ps for TA modes with an energy of 4 meV at 300 K, smaller but close to our findings, and a lifetime of about 1 ps for optic modes. It results that most of the heat carrying phonons have simulated mean free paths in the range 2 to 20 nm at 300 K, similarly to our case.

The case of Bi_2_Te_3_ seems much more similar to ours. Indeed in this system the acoustic regime is restricted to the range 0–6 meV, followed by weakly dispersive optical excitations in the range 6–10 meV and dispersionless optical modes in the range 10–16 meV. In fact this system bears some similarities with our clathrate, with an acoustic energy cut-off at about the same value of 6 meV, despite a sound velocity about 1.7 times smaller. Moreover, the heat carrying phonons have mean free paths in the range 7 to 70 nm, again very similar to our findings (see ref. ^62^).

Despite the similarities between these and our system, in PbTe and Bi_2_Te_3_ the thermal conductivity changes as 1/*T* at high temperature, in perfect agreement with a dominating rule of three –phonon processes, well reproduced in DFT simulations^8^. Moreover in the clathrate system the phase space available is also somewhat smaller than in the above two, since the heat carrying phonons only exist in the acoustic regime for the 0–4 meV energy range.

In summary, we have performed a quantitative measurement of the acoustic phonon lifetimes in a clathrate Ba_7.81_Ge_40.67_Au_5.33_ crystal at 300 and 10 K. We find propagative heat carriers with long lifetime, travelling distances from more than hundred to twenty unit cells for wave-vectors between 0.1 and 0.3 Å^−1^. Considering only the low-energy acoustic phonons and the experimentally determined phonon lifetimes allows us to reproduce the room temperature lattice thermal conductivity. Our results provide a picture of thermal transport in these systems and dramatically underline the inability of state of the art simulations to reproduce the observed phonons lifetime and thermal conductivity, asking for theoretical developments, for which we provide here a crucial experimental input.

## Methods

### Material synthesis and characterization

The single crystal of Ba_7.81_Ge_40.67_Au_5.33_ for neutron measurements was grown by the Bridgman technique^41^. Its crystal structure was characterized by single-crystal X-ray diffraction. The bulk mosaicity, as determined by hard X-ray and neutron Larmor diffraction, is less than 1' (Supplementary Note 1). The thermal conductivity was measured between 4 and 300 K (Supplementary Note 3).

### Thermal conductivity measurement

The thermal conductivity measurements were carried out in a home-made device using a steady state heat flow method in the temperature range 4 K < *T* < 300 K. The temperature gradient along the sample was applied by a small-resistance-chip heater with a room-temperature resistance of 120 Ω. The temperature gradient along the sample was determined by a differential Au/0.07%Fe vs. Chromel thermocouple and the absolute temperature was measured by a Ge resistor below 40 K and a Pt resistor from 40 to 300 K. Three concentric radiation shields were mounted around the sample in order to reduce the radiation effect. The uncertainty of the thermal conductivity measurement is ~ 5%. Nevertheless, data above ~ 100 K were affected by the radiation. The radiation effect was subtracted by using thermal conductivity data measured above 80 K by means of the 3ω technique in a commercial nitrogen flow cryostat from Cryovac. This is an ac technique which heats the sample locally and thus reduces errors caused by radiation at room temperature and below to a negligible level^63^. A narrow metal line (20 μm wide and 1 mm long) serves as both the heater and the thermometer. To avoid electrical contact between heater and sample the polished sample surface is covered with a thin layer of SiO_2_ by chemical vapour deposition^64^. More details on used 3ω setup can be found in ref. ^64^.

### Transmission electron microscopy

The Dresden Grand ARM (double corrected JEM-ARM300F) microscope was used for aberration-corrected TEM and STEM imaging at 300 kV^65^.

### Neutron scattering

Neutron measurements were performed on time-of-flight (TOF) and triple axis spectrometers (TAS) at the Institut Laue Langevin (IN5@ILL and IN22@ILL, Grenoble, France) and at the Laboratoire Léon Brillouin (2T@LLB, Saclay, France; for instruments and analysis details see Supplementary Note 4). The LA and TA dispersion and phonon lifetimes in Ba_7.81_Ge_40.67_Au_5.33_ were measured around the strong Bragg peak (600) at *Q* = 3.6 Å^−1^ in the scattering plane [100]-[011]. The instrumental resolution model when measuring dispersive excitation, calculated within the “affit” program, was carefully checked using a Ge single crystal (Supplementary Note 4). The experimental data have been then fitted using an excitation model (Damped Harmonic Oscillator: DHO) convoluted by the instrumental resolution (as detailed in ref. ^29^). The NRSE technique, on the TAS spectrometer IN22 at ILL (“ZETA” option), was used to determine phonon lifetimes up to 66 ps (for details see Supplementary Note 4).

### Theoretical calculations

DFT ab initio calculations with the VASP and PHONON codes were used to simulate the p-DOS, phonon dispersion, INS phonon cross section and the Au/Ge disordered configurations on supercells with sizes 2 × 2 × 2 (Supplementary Notes 2, 5 *)*. Simulations of phonon lifetimes in Ba_8_Ge_40_Au_6_, by considering cubic anharmonicities and the resulting phonon-phonon scattering, were performed using the ALAMODE code (Supplementary Note 5).

### Data availability

The authors declare that the data supporting the findings of this study are available within the article and its Supplementary Information file.

## Electronic supplementary material

Supplementary Information
