# Supplementary material for: Direct measurement of individual phonon lifetimes in the clathrate compound Ba7.81Ge40.67Au5.33
Source: Nat Commun. 2017 Sep 8;8:491. doi: 10.1038/s41467-017-00584-7 (PMC5591192; doi:10.1038/s41467-017-00584-7)
Supplement: Supplementary file 1 — Supplementary Information [file 41467_2017_584_MOESM1_ESM.pdf]

Description of Supplementary Files

File Name: Supplementary Information
Description: Supplementary Figures, Supplementary Tables, Supplementary Notes and Supplementary References

Supplementary Note 1: sample growth and characterization

Synthesis and primary characterization

As starting material for the crystal growth experiment, 20 g of polycrystalline sample with nominal composition $Ba_8Au_{5.25}Ge_{40.3}$ were prepared using as elemental components Ba pieces (Alfa Aesar, 99.9% metal basis), Au pieces (Chempur, 99.999% metal basis), and Ge pieces (Chempur, 99.9999 %). First, the binary phase BaGe was synthesized as an educt to avoid a vigorous exothermic reaction on melting. To obtain BaGe, the elements were molten in closed Ta ampoules and cooled down to room temperature within 3 minutes. Then, a total amount of 18 g BaGe (pieces), Au (pieces) and Ge (powder <200 μm) were molten in stoichiometric ratio under argon atmosphere in an open glassy carbon crucible (Sigradur, HTM) and cooled down within several minutes. After this process, the sample did not show any measurable mass loss. The precursor product was ground to powder with particle size < 500 μm and placed in a glassy carbon crucible with a narrow tip at the bottom. The sample was placed under argon atmosphere in a Bridgman furnace and heated to 980 °C within 48 h and kept at this temperature for another 24 h. In the next step the furnace was moved upwards for 8 cm within 10 days. After this time, the bottom of the growth crucible had a temperature of about 500 °C. The crucible showed no obvious traces of a reaction with the reaction mixture. The specimen obtained had a diameter of 10 mm and a length of 18 mm. The optical microstructure analysis clearly showed the presence of large single-crystalline grains in the product. According to the X-ray characterisation, the specimen contained three such single crystalline grains. The largest one (Supplementary Figure 1, panel a) was used for neutron scattering experiments. The remaining part was then shaped using a wire saw in appropriate specimens for further investigations.

The material was further characterized by the Guinier technique (Huber image plate camera G670, Cu $K\alpha1$ radiation, λ = 1.54056 A, measured region 3° < 2θ < 100°, step width 0.005°) using LaB_6 (a = 4.1569 Å) as an internal standard. A unit cell parameter of a = 10.7987(1) Å was determined by means of a least-squares refinement with the program package WinCSD (For details concerning WINCSD see http://wincsd.eu). Chemical analysis (WDSX) gave a Ba/Au ratio of 1.43(2) ≈ 7.81(4): 5.34(4), in good agreement with the results of the crystal structure determination.

The single grain crystalline quality

The ability to measure a phonon lifetime of the order of several tens of picoseconds by means of neutron spectroscopy requires a large, high-quality, single grain crystal. A large sample is required for the use of a very high resolution, low throughput experimental setup, so that the phonons linewidth is limited by the crystal mosaicity. We succeeded in growing such a large (~1.5 cm^3) single grain of $Ba_{7.81}Ge_{40.67}Au_{5.33}$, shown in Supplementary Figure 1, panel a, with a bulk mosaicity less than 1', as determined by hard X-ray and neutron Larmor diffraction.

A hard X-ray diffraction pattern of our single crystal $Ba_{7.81}Ge_{40.67}Au_{5.33}$ was measured at ILL (λ=0.027 Å) in order to determine its bulk mosaicity. The use of high energies allows for measuring centimeter-sized samples in transmission geometry. This method[1] allows disorientation of the atomic planes to be detected with a resolution better than 0.01°. Supplementary Figure 1 panel b shows the diffraction image as measured in the plane [110]-[011] of our sample. A cut is taken along a given direction, for example the [110] direction in the figure, and the profile is fitted with a Gaussian function (Supplementary Figure 1, panel c), whose width gives the sample mosaïcity. Here it is of 0.011° independently of the direction probed ((100), (110) and (011)).

This result was confirmed by Neutron Larmor diffraction (NLD) measurements on the spectrometer IN22 at ILL[2]. NLD is a now well-established, powerful technique for high-sensitivity neutron diffraction studies, allowing for absolute measurements of the lattice constants and mosaicity (for details on the technique see [[3],[4]]). The measurement of the final beam polarization $P_f(\varphi_{tot})$ as a function

of the Larmor phase at the Bragg position (6, 0, 0) in $Ba_{7.81}Ge_{40.67}Au_{5.33}$ is shown in Supplementary Figure 1, panel d. The value of the mosaicity extracted from this measurement is equal to 0.03° which corresponds to the resolution limit of the spectrometer as shown by direct comparison with measurements taken on a pure, high-quality, single crystal of Si.

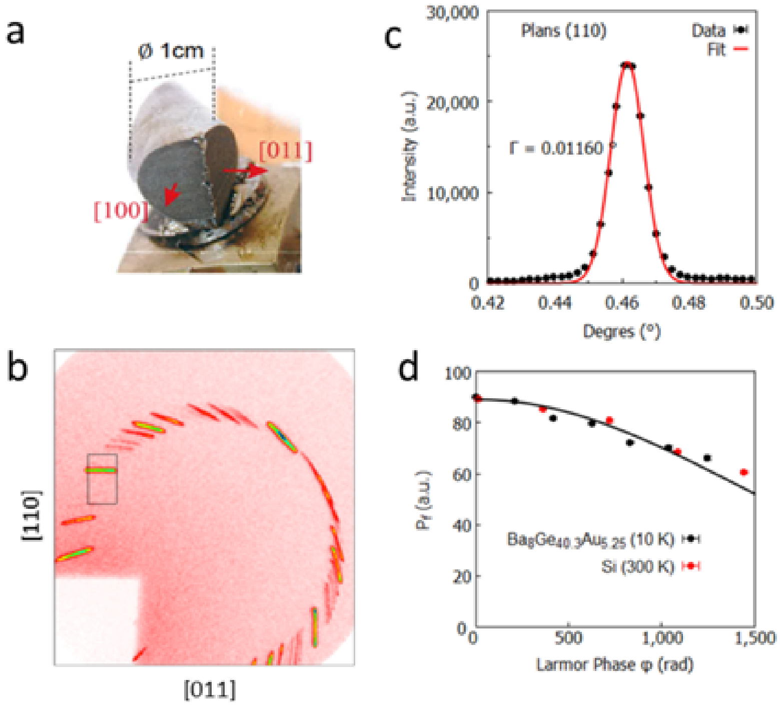

Supplementary Figure 1: Crystalline quality of the $Ba_8Ge_{40.3}Au_{5.25}$ single crystal used for neutron scattering. a) *Photograph of the single crystal of $Ba_8Ge_{40.3}Au_{5.25}$ used in the INS experiment.* ***(b-c)*** *Hard X-ray diffraction image for the [110]-[011] plane. The black squares indicates in c) indicate the cut along the direction [110], whose profile is reported in c) (black dots) with a Gaussian fit (red curve). The width of the Gaussian gives the sample mosaïcity (Γ).* ***(d)*** *Neutron Larmor diffraction measurements in $Ba_8Ge_{40.3}Au_{5.25}$ (black points) at the Bragg (6, 0, 0) and in Si (red points) at the Bragg (4, 4, 4). The final beam polarization, P_f, is shown as a function of the total Larmor phase (φ_{tot}). Data in $Ba_8Ge_{40.3}Au_{5.25}$ are fitted with the function $P_f(\varphi_{tot})$ (solid black line) in order to extract the sample mosaic*

Supplementary Note 2: crystal structure determination and simulation

Experimental structure determination

An irregularly shaped (150×150×190 μm) fragment of the single crystal was used for the diffraction experiments (Rigaku AFC7, Saturn724+ CCD detector), Mo$K\alpha$ radiation, λ = 0.71073 Å). 11876 reflections were measured, 8599 reflections observed with $I > 2\sigma(I)$, 711 of them were symmetry independent and used for structure solution and refinement. The final structure model is: space group $Pm\bar{3}n$, a = 10.7987(1) Å; $2Ba_1$ in 2(a) 000; 5.81(4)Ba_2 in 48(l) 0.2404(2) 0.5209(2) 0.0063(3); 5.33(4) Au in 12(g) 0.2591(1) 0 ½; 0.70(5)Ge_1 in 6(c) ¼ 0 ½; 16Ge_2 in 16(i) 0.1833(1) x x; 24Ge_3 in 24(k) 0 0.30870(9) 0.11796(8); $R(F)$ = 0.039 for the observed symmetry independent reflections and 0.035 for all observed reflections. The final refined composition is $Ba_{7.81(4)}Ge_{40.67(4)}Au_{5.33(4)}$. The Ba/Au ratio is in good agreement with the results of chemical analysis. In the manuscript, the composition of the single crystal investigated is given as $Ba_{7.81}Ge_{40.67}Au_{5.33}$.

A first interpretation of the disorder at the atomic level is a result of the tendency for covalent Ba–Au bonding. In a hypothetical binary model Ba_8Ge_{46}, the Ba2 atoms would be located in the centre of the 24-vertice polyhedron formed by Ge atoms and have 4 Ge1 atoms at the distances of 3.82 Å (Supplementary Figure 2, panel a). Substitution of Ge1 by Au leads to the Ba2–Au bonding and results in

a shift of the Au and Ba atoms within the Ba2 polyhedron (Supplementary Figure 2, panel b). With the partial occupation of 88.4% of Au at the Ge1 position, most Ba2 polyhedrons contain three or four Au atoms. In case of three Au atoms, Ba2 atoms are shifted from the 6*d* position ¼ ½ 0, so that the Ba2-Au distances are much shorter as Ba2-Ge1 (Supplementary Figure 2, panel b). A possible local configuration of the Ba2 polyhedron with four Au atoms is characterized by three atoms shifted in one direction, whereas the fourth one is shifted to the opposite one (Supplementary Figure 2, panel c). In this configuration, the Ba2-Au distances are closer to each other as in case of the three Au atoms, but the Ba2 atoms are located out of the 6*d* position. Characteristic lengths l were calculated by using the relation $l = a \times \sqrt[3]{\frac{100\%}{occ.}}$, where occ. is the occupancy coefficient (in %) for the minority component (defects) at a given site and a is the lattice parameter.

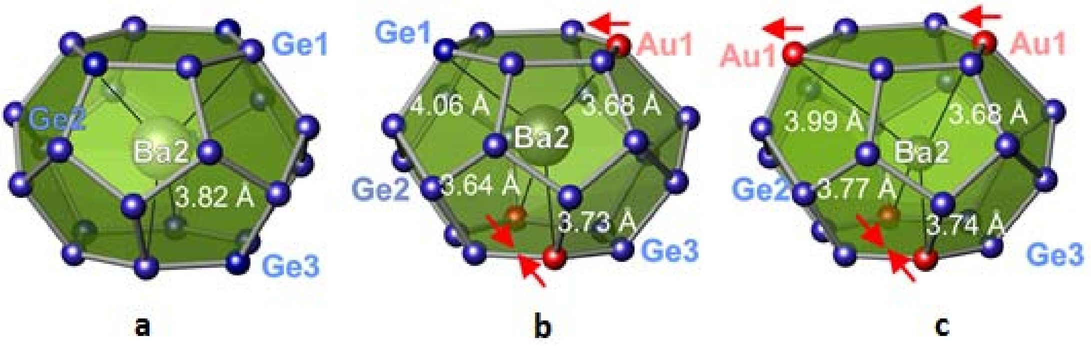

Supplementary Figure 2: Experimental crystal structure of $Ba_{7.81}Ge_{40.67}Au_{5.33}$. a*) Ideal ordered arrangement in the Ba2 polyhedron with Ba2 in 6d site and four equivalent Ba2-Ge1 distances.* ***b****) Local atomic arrangement in the Ba2 polyhedron with three Au atoms at Au1 site, Ba2 displaced from the 6d site and reduced Ba2-Au1 distances due to the Ba-Au interactions.* ***c****) Alternative local arrangement in the Ba2 polyhedron with four Au atoms at Au1 site, Ba2 displaced from the 6d site and reduced Ba2-Au1 distances.*

We studied the energetic stability as a function of the composition of the 6*c* site, at T = 0 K, in a (2x2x2) supercell. At finite temperatures entropy of chemical mixing may modify the stable composition range.

Energetic stability

For a moment, let us assume that the site 6*c* is fully occupied by Au, yielding the composition $Ba_8Ge_{40}Au_6$. From DFT calculation, the ternary compound is robustly stable against decomposition into competing binary phases: its total energy is safely (by 62 meV/atom) below the composition-energy tie-plane defined by Ge, $GeAu_2$ and $BaGe_2$ phases (binary clathrate Ba_5Ge lies just near the tie-line Ge-$BaGe_2$ and subtle questions arise regarding its low-temperature structure). The Au site occupancy is strictly limited to the 6*c* site: adding an Au on either of the other Ge sites costs 0.4 eV, and swapping Au/Ge from a 6*c* site to a 16*i* or 24*k* nominal Ge site costs 0.6 eV. The next paragraph describes the effect of depleting Au from $Ba_8Ge_{40}Au_6$ by fractionally occupying the 6*c* (Au) site by Ge atoms.

Ge occupancy on 6c site: energetic picture at zero temperature

Models with n Ge atoms on the Au-6*c* site were constructed by randomly placing every Ge atom on "6c" sites in the supercell. Let us note that the 6*c* atoms form large elongated tetrahedra with 5.5 Å and 6.7 Å edge lengths around Ba_2 atoms occupying centers of large 24-atom cages, while the smaller 20-atom cage around Ba_1 is strictly made of Ge. Correlations in distributing Ge atoms on the 48 6*c* sites in the (2x2x2) supercell were found negligibly weak.

Experimentally, the Ge content on Au 6*c* site is 11%, i.e. 0.67 Ge atoms per unit cell. For our study, we work with a (2x2x2) supercell holding 8x54=432 atoms. In this supercell, the 6*c* site orbit is represented by 48 Au atoms. We gradually replace Au atoms by Ge *one by one randomly*, compute DFT total energies, and evaluate zero-temperature stability as a function of Ge content on the 6*c* site. This is done by calculating the convex hull of mixing enthalpies for all known binary and ternary compounds, as well as the corresponding elements (notably Ge; see ref.[5]) as shown in Supplementary Figure 3. The red line is the trace of the tie plane drawn from the enthalpy of formation of known stable phases. As seen in Supplementary Figure 3, this plane arrives tangentially when 5 over 48 Au atoms are replaced by Ge, which indicates that the phase is stable when the number Au substituted atom is smaller or equal to 5 (10% Ge), and slightly unstable for 6 substituted Au atoms, in good agreement with what is known experimentally.

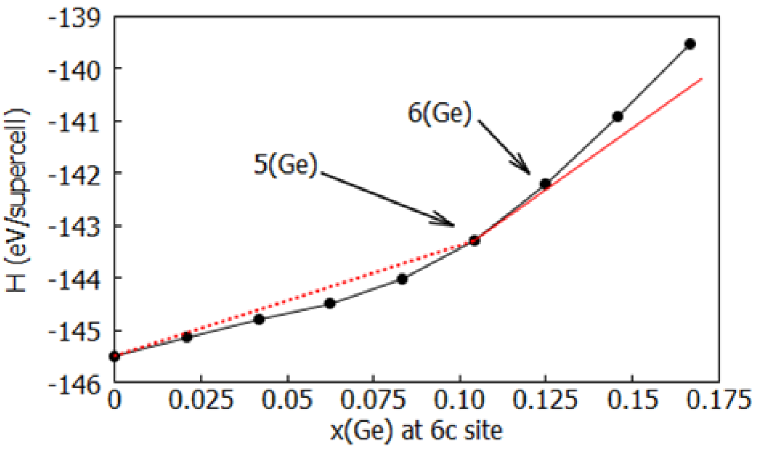

Supplementary Figure 3: Zero temperature stability of clathrate as a function of the fraction on the Au(6c) site. *Black circles represent mixing enthalpies of (2x2x2) supercell models, with 0-8 Ge atoms at Au(6c) over a total of 48 atoms. Up to "5 Ge" concentration, the phase is stable, becomes slightly unstable for "6 Ge". Red line indicates section through tie-plane of known stable phases, reaching at x=1 a mixture of $BaGe_2$ and Ge phases.*

Local atomic configuration

We have studied samples of the 2x2x2 supercell model, with independent random substitutions of 5 Ge atoms at the Au site, in the P1 space group and a lattice parameter equal to a = 1.09874 nm. Because of the partial Au occupation of the 6*c* site, we find two main configurations for the large cage:

(i) About 40% of the cages have only 3 Au atoms forming a triangle, almost equilateral. In this configuration we find that the Ba2 atoms are displaced in the direction of the Au triangle, with a vector (in Å) equal to (0.11636 0.23581 0.00000), d=0.26 Å, in very good agreement with the experimentally determined one equal to (0.086448, 0.20, 0) d=0.21 Å. In this case it is also found that two out of three atoms stay at the ideal 6*c* position, whereas one Au atom moves outward by 0.07 in good agreement with the 0.08 Å displacement found in the experimental splitted site. The Ge atom remains on the ideal 6*c* position, again in good agreement with the experimental atomic structure.

(ii) The remaining 60% have 4 Au atoms, as in the ordered state. In this case we find that the Ba2 atoms is also displaced from the cage centre by about 0.07 Å. About half of the Au atoms are displaced (both direction) by 0.07 Å, whereas the other half stays at the ideal 6*c* position.

It has to be noticed that a few sites have only 2 Au atoms in the large cage.

Supplementary Note 3: Lattice thermal conductivity

As predicted by *ab-initio* calculations, the lattice thermal conductivity of $Ba_{7.81}Ge_{40.67}Au_{5.33}$ is found to be lower than in lighter transition metals substituted Ge clathrates, being only 1.1 $W.m^{-1}.K^{-1}$ at room temperature, as found in[6,7,8]. In Supplementary Figure 4 we report its temperature dependence as measured on a single crystal between 4 K and 250 K and compare it with the lattice thermal conductivity of pure Ge, to highlight the dramatic effect of the clathrate structure on thermal transport. Not only the thermal conductivity is reduced by one to two orders of magnitude, but also the Umklapp peak is smoothed out, as observed also in other clathrates, reminiscent of the well-known plateau in amorphous systems and quasicrystals (long range ordered structures without lattice translation symmetry).

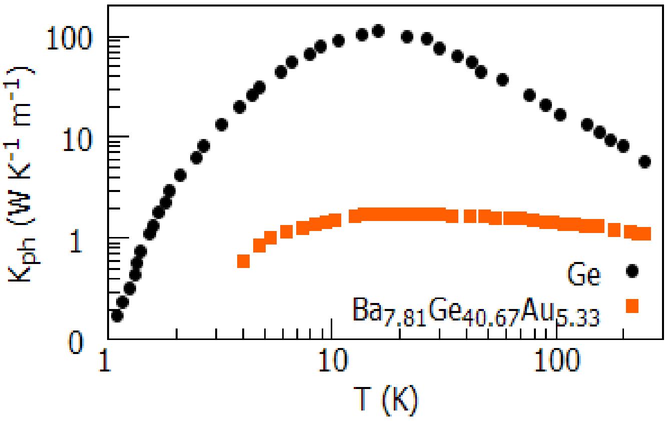

Supplementary Figure 4: Lattice thermal conductivity. *The lattice thermal conductivity of $Ba_{7.81}Ge_{40.67}Au_{5.33}$ measured along the direction (111) (red squares) is compared with the one of Ge (black dots). The Ge curve corresponds to data from et Chung J. D. al[9] divided by a factor of 10 for better comparison with the clathrate.*

Supplementary Note 4: Inelastic neutron scattering measurements

Triple axis spectrometer (TAS) measurement

The triple-axis 2T@LLB[10] spectrometers was used to investigate the TA dispersion in $Ba_{7.81}Ge_{40.67}Au_{5.33}$ around the Bragg peak (600) at Q = 3.6 Å^{-1} in the [100]-[011] scattering plane (Supplementary Figure 6).

The measurements on 2T were performed using a pyrolytic graphite (PG002) analyser and monochromator and a fixed final neutron wave-vector of k_F = 2.662 Å^{-1}. A graphite filter after the sample suppresses higher order contaminations. We collected data using "standard" and "high-resolution" experimental settings. In the former, a vertically and horizontally focussing PG002 analyser was used with no collimation giving an energy resolution of 1.14 meV (FWHM) in the energy range 0-20 meV, as measured on the elastic incoherent scattering of Vanadium. In the high-resolution setup, a flat monocrystalline PG002 analyser was used, with horizontal collimations 20'-20' and vertical slits (after the sample), resulting in an energy resolution of 0.84 meV.

TAS resolution for dispersive excitations

For dispersive excitations defined by a surface $\omega(\mathbf{q})$ in phase space, the observed peaks result from the convolution of the 'phonon surface' with the resolution, which is itself a function of ω and $\mathbf{q}$. Supplementary Figure 5, panel a shows the simulated instrumental energy resolution as a function of the local slope (in meV Å^{-1}) at Q = (6, 0.2, 0.2) and ω = 5 meV, close to our experimental conditions, for the two TAS settings. It can be seen that the best resolution is achieved at a slope of 11 meV.Å^{-1} (as in the case of the $Ba_{7.81}Ge_{40.67}Au_{5.33}$ clathrate at this wave vector), resulting in a FWHM of 0.2 meV for the high-resolution setup.

In order to confirm the simulated instrumental resolution, we have measured the transverse branch of a pure Ge single crystal TA_{100}^{011}, around the Bragg Peak (022), where the slope is also 11 meV Å^{-1}. Supplementary Figure 5, panel b reports data collected at Q = (0.38 ,2 ,2) compared with the simulated phonon spectrum assuming no broadening for the phonon mode (modelled with a Dirac function) and the instrumental resolution calculated for this slope[11]. The perfect agreement allows us to consider the Ge data as representative of the TAS instrument resolution contribution.

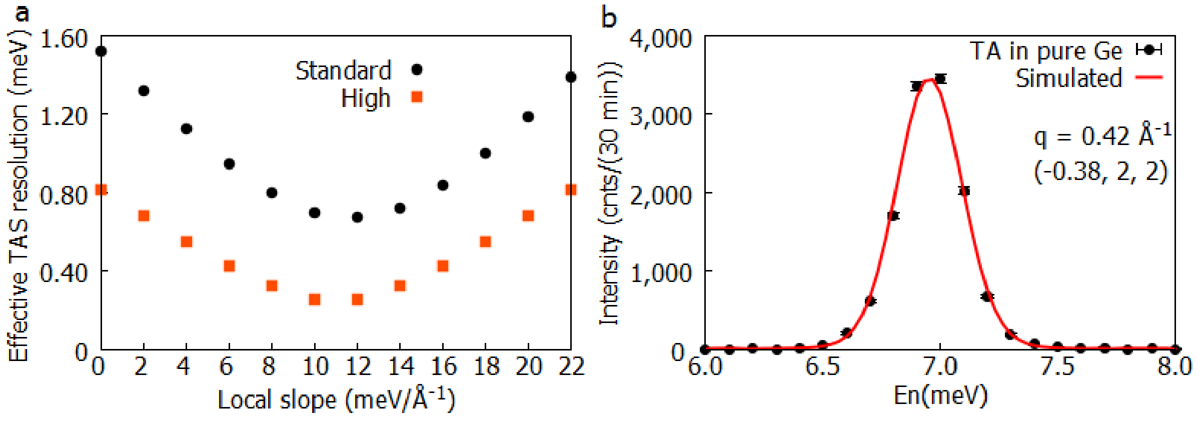

Supplementary Figure 5: TAS effective resolution on 2T@LLB. a) *Instrument resolution with a standard (black curve) and high resolution setup (orange curve) as a function of the slope of the acoustic branch.* ***b)*** *Experimental data (black dots) and simulated resolution function (red curve) on a pure Ge crystal measured with the high resolution set-up: there is a perfect agreement.*

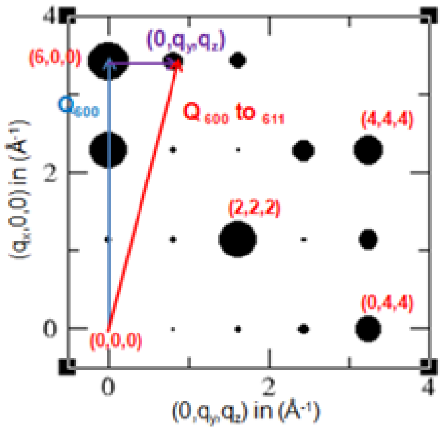

Supplementary Figure 6: Reciprocal space Bragg peak intensity distribution and geometry used for the TAS measurement in the [100]-[011] plane. *Bragg peak intensities have been calculated for a neutron wave vector* k_F = *2.662* Å^{-1}. *The point area is proportional to the nuclear elastic structure factor.*

Using the resolution model function we have extracted from the HR TAS measurements the position and width of the TA excitation in the clathrate, as shown in Supplementary Figure 7. The presence of the low-lying excitation at 4.5 meV reduces the accuracy of the extracted TA phonon width. Two fitting procedures have been used, giving different intensity weight to this optical excitation, from which error bars have been extracted (Figure 4 main text). Only one fitting procedure is represented in Supplementary Figure 7.

For higher energy optical excitations measured with the standard resolution set up (noted E2, E3 and E4 in the main text), the measured excitation is broader than the experimental resolution (see Figure 3,

main text). This might be due to the superposition of several weakly dispersive modes, or a single one with finite lifetime. Under the assumption of a single excitation, the optical excitations have a lifetime of the order 1 ps.

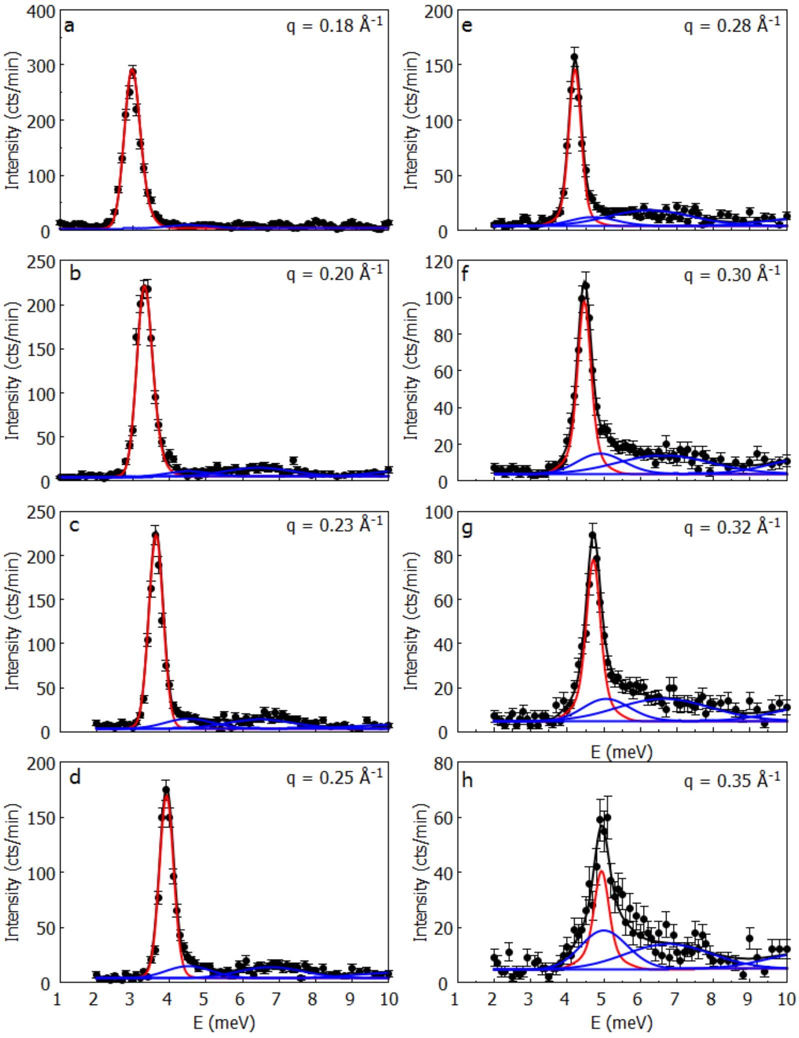

Supplementary Figure 7: Constant Q energy scans of TA excitations using the HR TAS set up 2T@LLB. *The dot black curve is the data and the black line is the result of the fit, whereas red and blue lines indicate the individual contributions. The excitation at about 7 meV, was fitted with the standard resolution set up and its position and width kept fixed during the HR data fit.*

Time-of-Flight inelastic neutron scattering measurements

We used the Time-of-Flight (TOF) spectrometer IN5@ILL[12] on the same clathrate single crystal. This allows a full mapping of the q-E space. We used an instrument setup with an incident wavelength of 3.2 Å giving an energy resolution (FWHM) of 0.27 meV at the elastic line, of 0.41 meV and 0.58 meV at the energy transfer of 5 meV and 10 meV on the anti-Stokes side and 0.18 meV on the maximum energy transfer of 5 meV on the Stokes side[13]. Rotating the sample by 1° steps, up to 69 spectra were collected and analysed using the Horace software[14] and covering a broad region.

Data are stored in a 4D matrix as a function of the three directions in reciprocal space and energy transfer. Figure 2 panels a and b in the main text displays 2D-intensity maps in the scattering plane, [00l],[hh0], which have been extracted from this 4D matrix. With this setting the phonon energy resolution remains extremely good, even in the longitudinal direction.

The dispersion relations of the acoustic branch, extracted from both TAS and TOF measurements are displayed in Supplementary Table 2 and Supplementary Figure 8.

	**Branch LA_{100}**	**Branch TA_{011}^{100}**
**Dispersion relation (E in meV and q in Å^{-1})**	$E(q) = 29.73 \times q - 30.24 \times q^2$	$E(q) = 17.86 \times q - 13.44 \times q^2$
**Sound velocity (m.s^{-1})**	V_{LA} = 4,520(56)	V_{TA} = 2,930(70)
**Elastic modulus (GPa)**	$C_{11} = (V_{LA})^2 / \rho$ = 135(4)	$C_{44} = (V_{TA})^2 / \rho$ = 41(3)
**Mean sound velocity (v_m) : $3v_m^{-3} = v_{LA}^{-3} + 2v_{TA}^{-3}$, $v_m = 3,000(75)$ m.s^{-1}**		

Supplementary Table 2: Elastic properties. *Dispersion relations, phonon velocities and elastic moduli in the clathrate $Ba_{7.81}Ge_{40.67}Au_{5.33}$, as determined from TOF and TAS measurements.*

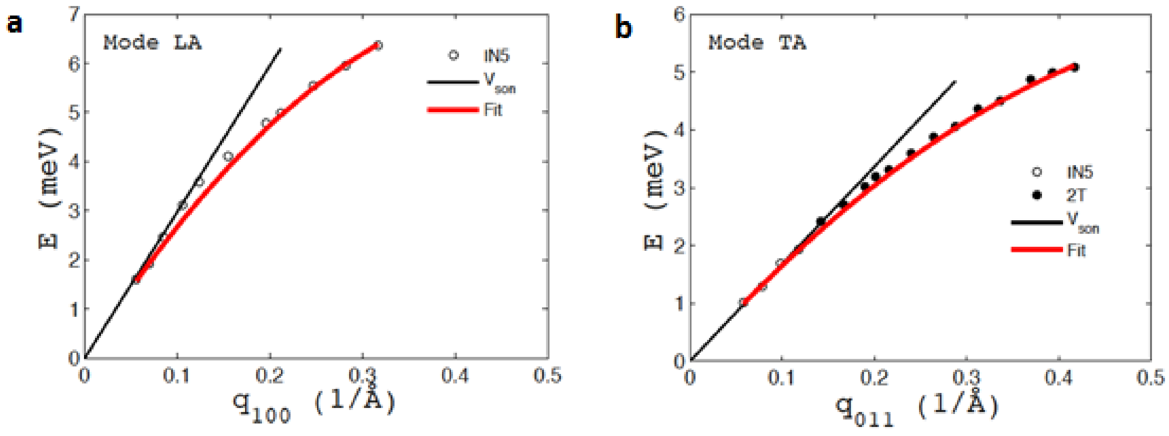

Supplementary Figure 8: The dispersions relations of longitudinal acoustic (LA) phonons in panel a) and transverse acoustic phonons (TA) in panel b) as extracted from triple axis (full black circles) and time-of-flight (open circles) measurements on the spectrometers 2T and IN5 respectively. *The solid black line corresponds to the extrapolation of the sound velocity at the center of the Brillouin zone and the red line to a fit to the dispersion.*

Neutron Resonant Spin-Echo measurements

The Neutron Spin Echo (NSE) technique coupled to a TAS spectrometer for measuring the lifetime of excitations gives a very high energy resolution in the µeV range[15,16]. For dispersive excitations, like acoustic phonons, a special focusing technique is necessary[15] ("spin-echo phonons focusing"), which consists of aligning the instrumental resolution function with the gradient of the dispersion surface. The Neutron Resonant Spin Echo (NRSE) technique[16,17] is a recent variant of NSE, which uses a combination of static and radio-frequency rotating magnetic fields – a resonance spin flipper, resulting in a spin phase change twice as large as the one obtained in NSE and leading to an improvement of the resolution. For our measurements we used the NRSE technique, on the TAS spectrometer IN22 at ILL ("ZETA" option)[2].

In the data treatment the total instrumental resolution function $P_R(\vec{Q}, \tau_{NSE})$ was calculated, once the sample properties and the NRSE geometry were known, using the MATLAB code SERESCAL[18]. The

intrinsic polarization is therefore obtained by renormalizing the measured one by the following factors: $P(\tau_{NRSE}) = P_{MEAS}/[P_0 \,.\, P_R(\vec{Q},\tau_{NSE})]$,where P_0 is the instrument intrinsic polarization at τ_{NRSE}=0.

Supplementary Note 5: Phonons ab initio simulations

Simulation of the INS phonon cross section

To study the lattice dynamics in the clathrate system, DFT *ab-initio* calculations were performed with the VASP code[19] using the projector augmented wave method and the generalized gradient approximation (PAW-GGA). The unit cell was relaxed to the ground state using a k-point mesh (5×5×5) and a convergence criterion of residual forces of less than 10^{-5} eV/Å. The Hellmann-Feynman forces were then calculated after introducing symmetrically non-equivalent displacements of +/- 0.03 Å in the relaxed unit cell. These forces are given as an input to the PHONON code for the calculation of the dynamical matrix[20], the eigenvalues and eigenvectors of which describe the phonons of the system in the harmonic approximation.

The neutron scattering intensity is calculated as:

$S_{ph}\,(\vec{Q},\omega) = n(\omega)\frac{|F_D^i(\vec{Q})|^2}{\omega_{q,j}}\delta(\omega-\omega_{q,j})\delta(\vec{Q}-\vec{q}-\vec{G})$ where *n(ω)* is the Bose factor, $\vec{G}$ is the nearest reciprocal lattice vector and $\vec{q}$ the phonon wave vector so that $\vec{Q} = \vec{q} + \vec{G}$.

For the comparison with experimental data reported in the main part (Figure 2 in the main text), the calculated $S_{ph}\,(\vec{Q},\omega)$ was convoluted with a Gaussian resolution function with an energy width equal to the TAS resolution: the slope-dependent one for dispersive excitations, as explained above, and a constant width as determined with Vanadium for the non-dispersive optic branches. Similarly to other clathrates, a large rescaling of the energy by a factor 1.4 for acoustic modes and 1.2 for optical excitations was necessary to achieve good agreement between the calculated and experimental acoustic and low-lying optic branches. The Supplementary Figure 9 shows the comparison between the experimental dispersion relation and the simulation for the two scaling factors.

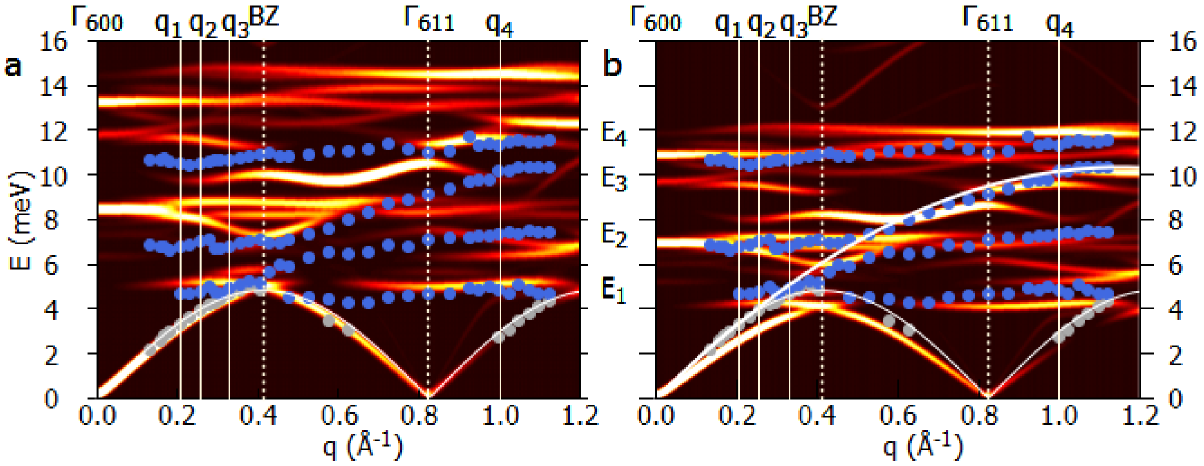

Supplementary Figure 9: Comparison between experiment and simulation. *Grey (blue) dots stand for acoustic and optical excitations as extracted from the TAS experiment. The background color coded picture stands for the DFT simulation.* ***a)*** *with an energy scaling factor equal to 1.4.* ***b)*** *with an energy scaling factor equal to 1.2.*

Effect of atomic substitution, on the Ge 6c site, on the phonon DOS

Prior to the detailed experimental and computational study of the $Ba_{7.81}Ge_{40.67}Au_{5.33}$ system, we have investigated the impact of substituting the *6c* position in Ba_8Ge_{46} clathrates by different transition metals (TM), similar to the study of S. Johnsen and *al.*[6]. Supplementary Figure 10 reports the comparison of the

calculated pDOS for three different TM clathrates $Ba_8Ge_{40}TM_6$ (with TM=Cu, Ag, Au). While the overall spectrum is similar, with distinct Ba modes at low energies and Ge modes at higher energies, the details strongly differ with the TM. The spectral range where the TM modes contribute most shifts towards lower energies from Cu to Ag and Au, which is due to the increasing atomic masses. In the case of the $Ba_{7.81}Ge_{40.67}Au_{5.33}$ compound, this shift results in an overlap of Ba- and TM modes, which leads to mode hybridization and thus decreases the lattice thermal conductivity[6,7]. The investigation of the lattice dynamics in $Ba_{7.81}Ge_{40.67}Au_{5.33}$ confirmed this scenario. As explained in the main text, above E_1, phonon spectra are dominated by optic modes, and can be modelled with four Gaussian distributions labelled $E_{1,2,3,4}$, which are the superposition of numerous optic modes. In comparison with previous INS studies on single crystals of $Ba_8Ge_{42.1}Ni_{3.5}$[11] and Ba_8Si_{46}[21], the peak E_2, containing mainly optical modes associated with the Au vibrations, comes between the E_1 and E_3 peaks associated with the Ba motions in the large and small cages respectively. As a result, E_1 = 4.5 meV is lower than in $Ba_8Ge_{42.1}Ni_{3.5}$ (5.5 meV) or in Ba_8Si_{46} (7 meV) while the peak E_3 is pushed to higher energies confirming the *ab-initio* prediction.

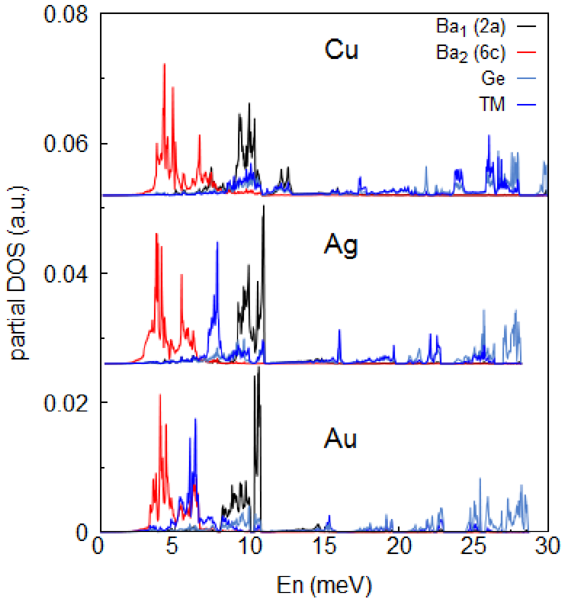

Supplementary Figure 10: The partial phonon density of states (pDOS) for different $Ba_8Ge_{40}TM_6$ clathrates, *as obtained by ab-initio simulations*.

Supplementary Figure 11 presents the simulated weighted partial density of states of the $Ba_8Ge_{40}Au_6$ clathrate. This figure allows assigning the atoms mainly involved in the E_1, E_2, E_3 and E_4 modes, namely Ba2, Au, Ge and Ge/Ba1.

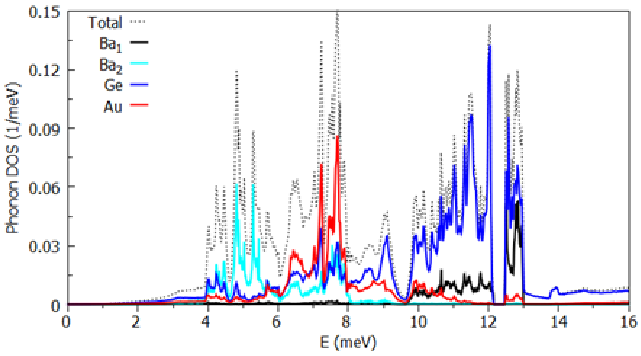

Supplementary Figure 11: The weighted partial phonon density of states (pDOS) of $Ba_8Ge_{40}Au_6$ clathrate, *as obtained by ab-initio simulations in the low energy region. The different atomic contributions are compared to the total vibrational density of state (black dashed line). For a direct comparison with experiment a scaling factor of 1.2*

has been applied to the energy scale. The experimental E_1, E_2, E_3 and E_4 optical band can be associated with Ba2, Au, Ge and Ge/Ba1 vibrations.

Three phonon transition probabilities in $Ba_{7.81}Ge_{40.67}Au_{5.33}$

Using the recently developed ALAMODE package of Tadano et *al.*[22], we have calculated the phonon lifetimes in $Ba_8Ge_{40}Au_6$ compound by considering cubic anharmonicity and the resulting phonon-phonon scattering. In this case we have used an ordered cubic cell with a composition equal to $Ba_8Ge_{40}Au_6$ and space group $Pm\bar{3}n$, i.e Au atoms fully occupy the 6c crystallographic site and the Ba atoms are located on the high symmetry positions. The harmonic force constants were calculated from 12 symmetry non-equivalent displacements of 0.02 Å in the optimised unit cell, whereas a set of 267 different configurations (containing multi-displacements of 0.04 Å each) was used to calculate the cubic force constants. All together, 320 harmonic and 384 cubic force constants were used to calculate the phonon lifetimes in $Ba_8Ge_{40}Au_6$. The resulting lifetimes for temperatures of 10 and 300 K are shown in Supplementary Figure 12, clearly demonstrating strong changes with temperature for 3-phonon scattering processes.

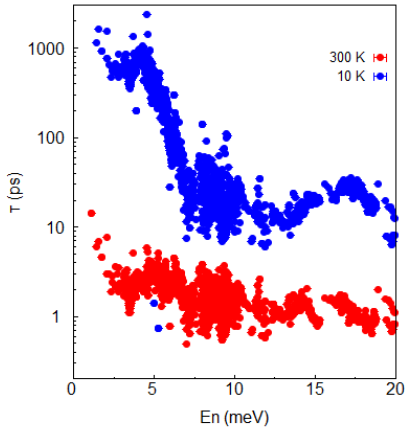

Supplementary Figure 12: Phonons lifetimes calculated for $Ba_8Ge_{40}Au_6$ using the ALAMODE package. *The phonon lifetimes at 10 and at 300 K are depicted in blue and red, respectively.*

As observed by Tadano et *al.*[22], we find that the phonons lifetime and corresponding thermal conductivity is very sensitive to the lattice parameter used in the simulation. If the RT experimental lattice parameter (equal to 1.085 nm) is used rather than the relaxed one (equal to 1.1 nm), we find an average increase of the phonon lifetime (and thermal conductivity) by roughly a factor 4 as shown in Supplementary Fig. 13.

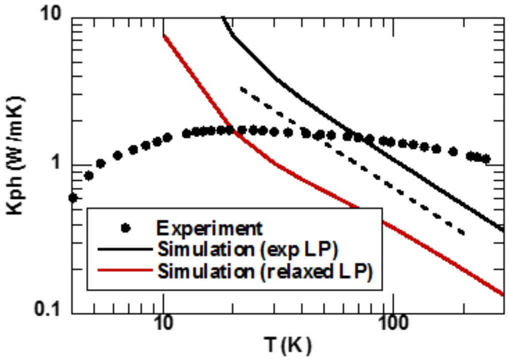

Supplementary Figure 13: comparison of the experimental thermal conductivity with the simulated one using the ALAMODE software *and the experimental lattice parameter (black curve) or the relaxed lattice parameter (red curve). The dashed line indicates a 1/T decay.*

When looking at the energy dependence of the acoustic excitations, an overall $1/E^2$ decay of the lifetime is roughly observed, although there is a significant anisotropy as shown in Supplementary Figure 14 for TA modes propagating along the (0 1 1) direction. In order to obtain a larger number of calculated points in the Brillouin zone, several k-space grids have been used and merged together (namely 5x5x5, 6x6x6 and 7x7x7).

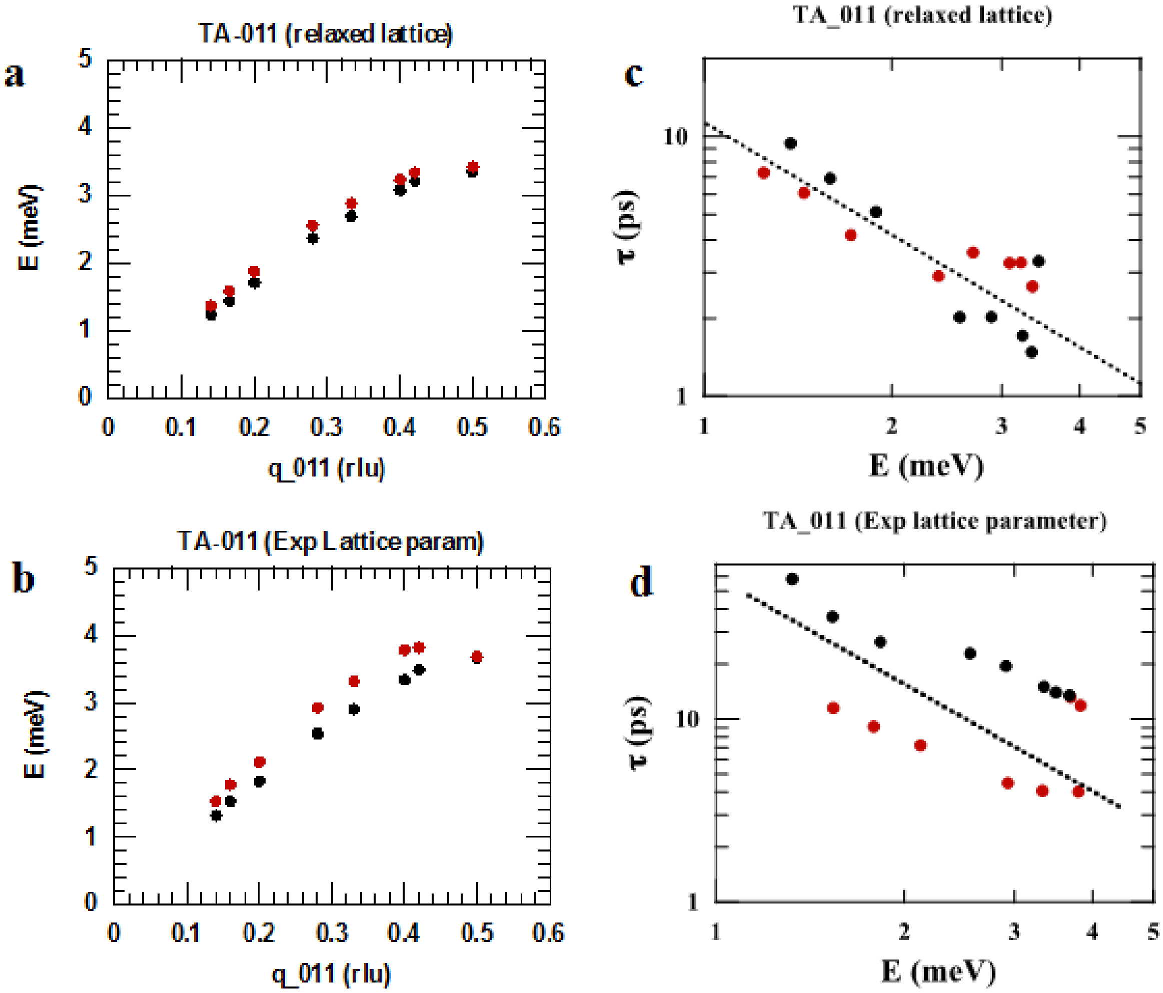

Supplementary Figure 14: Simulation of TA_{011} dispersion (panels a and b) and lifetime (panels c and d) using relaxed (panels a and c) and the experimental (panels b and d) lattice parameter. *The dashed line in the panels c and d indicates a $1/E^2$ decay. Red and black dots stand for the two polarizations of the TA modes respectively. Note the large change of the phonon lifetime for one of the mode when using the experimental lattice parameter.*

We have also tried to evaluate the effect of disorder on the calculated phonon lifetime. Current computer power capability prevents a simulation on a 2x2x2 unit cell. We have thus used a 1x1x1 unit cell, with a substitution of one Au atom by a Ge atom, leading to a compound having the composition $Ba_8Ge_{41}Au_5$ and a lower symmetry than the ordered one. As expected the disorder increases the 3 phonons scattering probability, leading to a thermal conductivity 3 times smaller.

As discussed in the article, the values found in the simulation are significantly different from the experiment.

Supplementary Note 6: Classical kinetic gas theory of thermal transport in complex systems

It has been long established that a good approximation of κ_L in complex dielectric crystals is obtained by assuming that only acoustic phonons are efficient for thermal transport, while neglecting optic phonons, although they mostly contribute to the heat capacity[23,24]. This approximation considers thus that optical excitations do not carry any heat, a hypothesis which is backed by the fact that those modes are generally almost dispersionless in complex systems. This approximation works especially well in guest-host materials where atomic masses and chemical bonds differ substantially, but its validity is a general characteristic of structurally complex systems, including non-periodic ones, like quasicrystals.

With this hypothesis only the acoustic modes, i.e. 3 vibrational modes, are taken into account for the thermal transport:

$$\kappa_L = \frac{1}{3} C\bar{v}l$$

With $\bar{v}$ the average sound velocity defined by $\frac{3}{\bar{v}^3} = \frac{1}{v_{LA}{}^3} + \frac{2}{v_{TA}{}^3}$ and deduced from the measured values v_{LA} and v_{TA}, here $\bar{v} = 3\ 000$ m.s^{-1} (see Supplementary Table 2).

There are several ways of deriving κ_L considering only the three acoustic branches. The most general approach, which applies to all systems including quasicrystals, is to consider that only 3 acoustic modes are contributing between 0 and E_{max}, where E_{max} correspond to the energy of the low lying optical mode (4.5 meV in our case). E_{max} can be viewed as a renormalized Debye temperature ($\widetilde{\theta_D}$).

In the high temperature regime, the heat capacity reaches the Dulong-Petit limit for only the three vibrational modes, equal to 3k$_B$T per unit cell, which for Ba-Au-Ge leads to C = 32584 J m^{-3} K^{-1}.

Using this normalized heat capacity, and assuming a mean free path of 25 nm, equal to the average value of our lifetime measurements for TA phonons with E ~ 3.2-4.4 meV, we find $\kappa_L^{ac}{\sim}1.0$ W·m^{-1}·K^{-1}, which accounts for more than 91 % of the total lattice thermal conductivity at room temperature. This confirms previous studies showing that in complex dielectric crystals κ_L is well approximated by considering only the acoustic phonons, even though the optic modes contribute mainly to the heat capacity, here more than 98 % (C_{DP}^{op} = 259.47 mJ·g^{-1}·K^{-1}), and provide scattering channels for acoustic phonons.

In the low temperature limit, the specific heat is expressed as a function of the renormalized Debye temperature $\widetilde{\theta_D}$. A trivial expression of $\widetilde{\theta_D}$ is obtained from the definition of the Debye temperature and the number of atoms in the unit cell N: $k_B\theta_D = \left(\frac{2(3N)\pi^2}{v_a}\right)^{1/3} \bar{c} = N^{1/3}\ \widetilde{\theta_D}$ where $\widetilde{\theta_D}$ is the Debye temperature for 3 vibrational modes only. For our crystal, the $\widetilde{\theta_D}$ estimated in this way is equal to ~ 82 K, i.e. 7 meV. Although the order of magnitude is correct, this value is slightly higher than E_{max}; the difference is likely due to the fact that the renormalisation by N takes into account only the structural complexity, missing the specific nature of the guest vibrations.

Moreover this approach breaks down for large unit cell or 'infinite' one like in quasicrystals, for it leads to a renormalized Debye temperature going to zero. We note however that for all intermetallic complex systems studied so far, including quasicrystals, a well defined acoustic regime is observed in a narrow q (0-0.4 Å^{-1}) and energy (0 to 5-7 meV[25,26,27]) range. We thus suggest using an experimentally renormalized Debye temperature $\widetilde{\theta_D}$ to calculate the lattice thermal conductivity *in all complex systems* including quasicrystals, as proposed by Takeuchi. This leads to similar mean free path of the order 20-30 nm.

Cumulative thermal conductivity.

Using the expression 1 (main text) for thermal conductivity calculation, the experimental phonons dispersions and the simple phenomelogical model describing the energy dependence of acoustic phonon

lifetime it is straightforward to calculate the cumulative thermal conductivity, which is displayed below, in Supplementary Figure 15 (panel a) together with the phonons density of states for the TA branch.

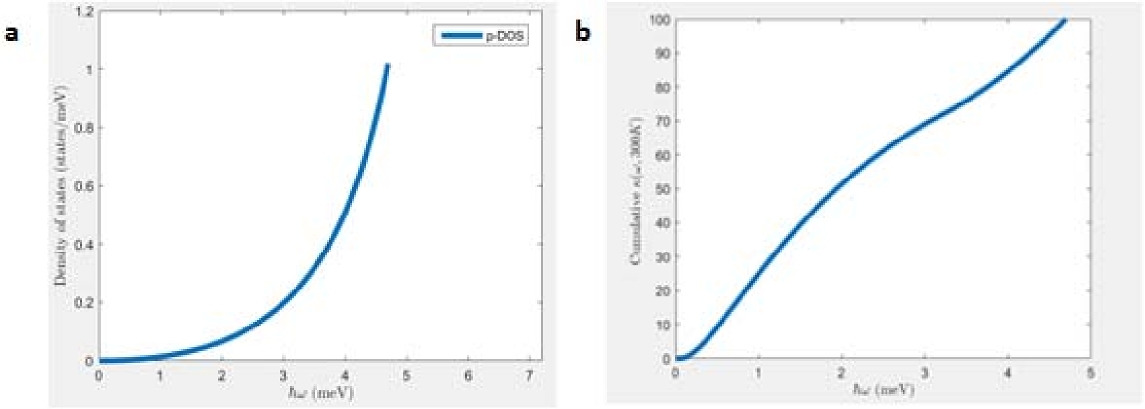

Supplementary Figure 15: a) Computed phonons density of states of the TA branch and b) Calculated cumulative thermal conductivity for $Ba_8Ge_{40}Au_6$. From the experimental dispersions and lifetimes. *About 50% of the thermal conductivity is carried by phonon in the range 0-2 meV, or the range 2-4.5 meV.*

Supplementary Note 17

The following pages display the data of the constant Q, energy scans as measured on the triple-axis 2T@LLB[10] spectrometers to investigate the TA dispersion in $Ba_{7.81}Ge_{40.67}Au_{5.33}$ around the Bragg peak (600). Dots are for experimental data and continuous lines for the fit. The red continuous line highlights the acoustic mode.

These data are available upon request to the authors.

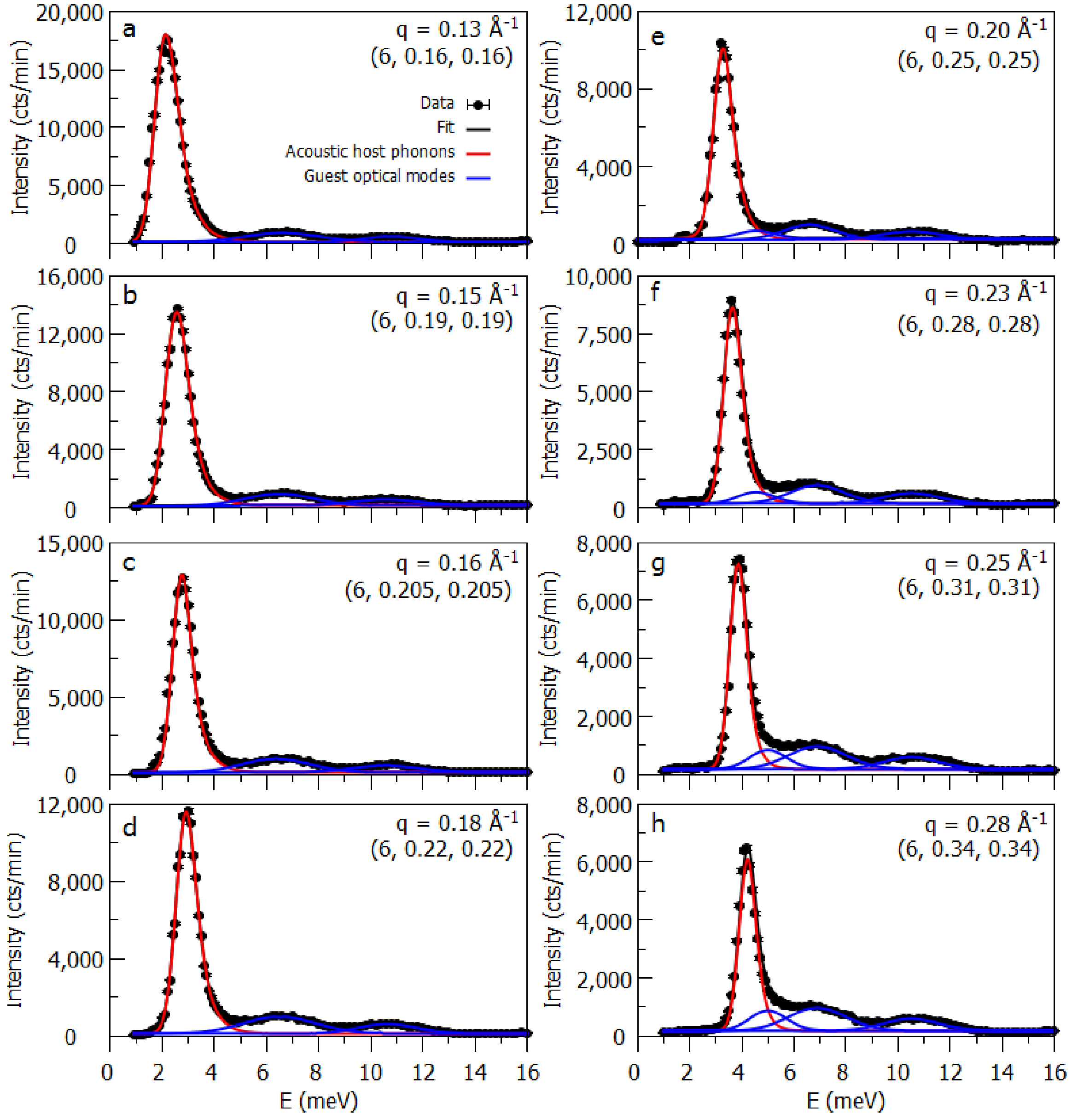

a
q = 0.13 Å^{-1}
(6, 0.16, 0.16)
Data
Fit
Acoustic host phonons
Guest optical modes
b
q = 0.15 Å^{-1}
(6, 0.19, 0.19)
c
q = 0.16 Å^{-1}
(6, 0.205, 0.205)
d
q = 0.18 Å^{-1}
(6, 0.22, 0.22)
e
q = 0.20 Å^{-1}
(6, 0.25, 0.25)
f
q = 0.23 Å^{-1}
(6, 0.28, 0.28)
g
q = 0.25 Å^{-1}
(6, 0.31, 0.31)
h
q = 0.28 Å^{-1}
(6, 0.34, 0.34)
Intensity (cts/min)
E (meV)

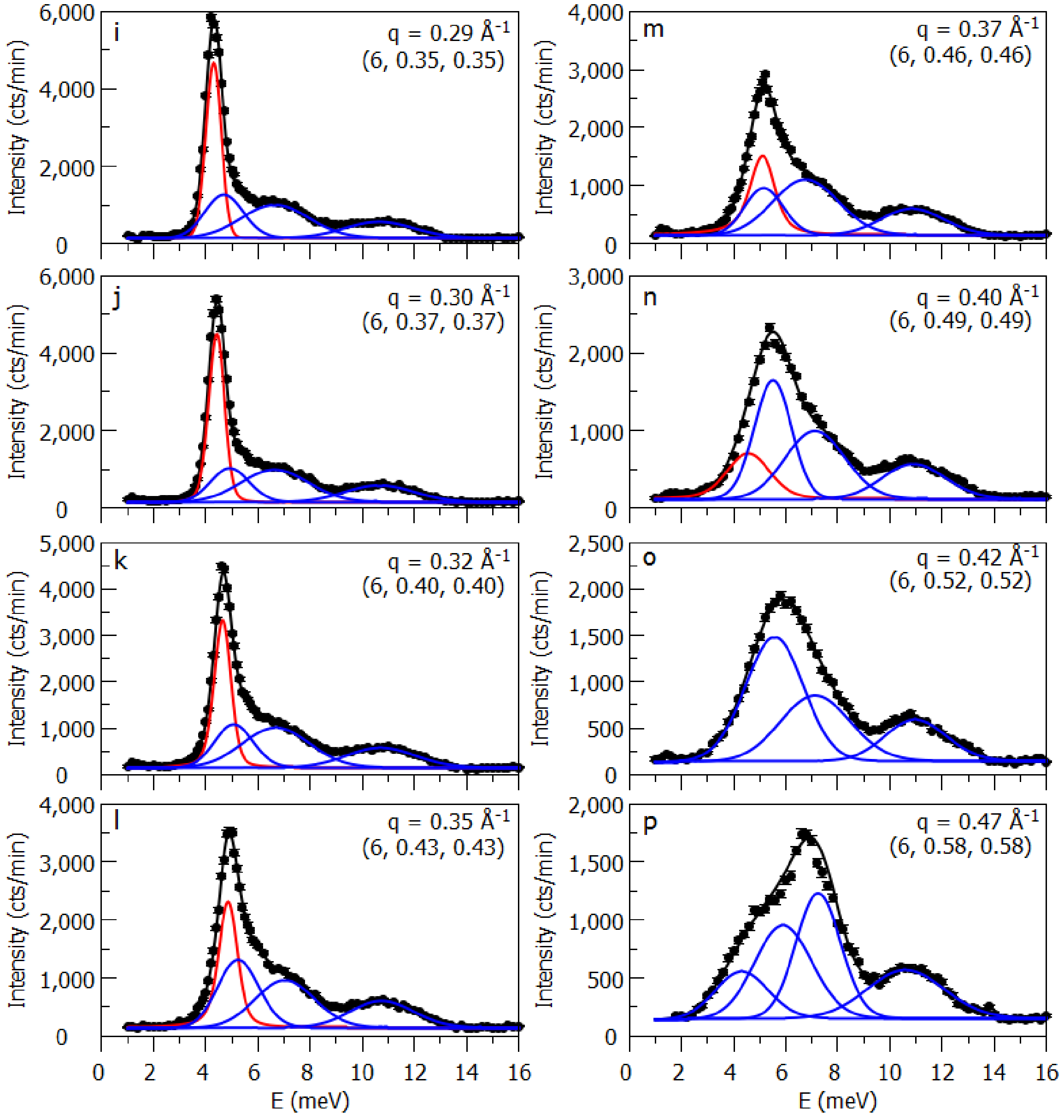
i
q = 0.29 Å⁻¹
(6, 0.35, 0.35)
j
q = 0.30 Å⁻¹
(6, 0.37, 0.37)
k
q = 0.32 Å⁻¹
(6, 0.40, 0.40)
l
q = 0.35 Å⁻¹
(6, 0.43, 0.43)
m
q = 0.37 Å⁻¹
(6, 0.46, 0.46)
n
q = 0.40 Å⁻¹
(6, 0.49, 0.49)
o
q = 0.42 Å⁻¹
(6, 0.52, 0.52)
p
q = 0.47 Å⁻¹
(6, 0.58, 0.58)
Intensity (cts/min)
E (meV)

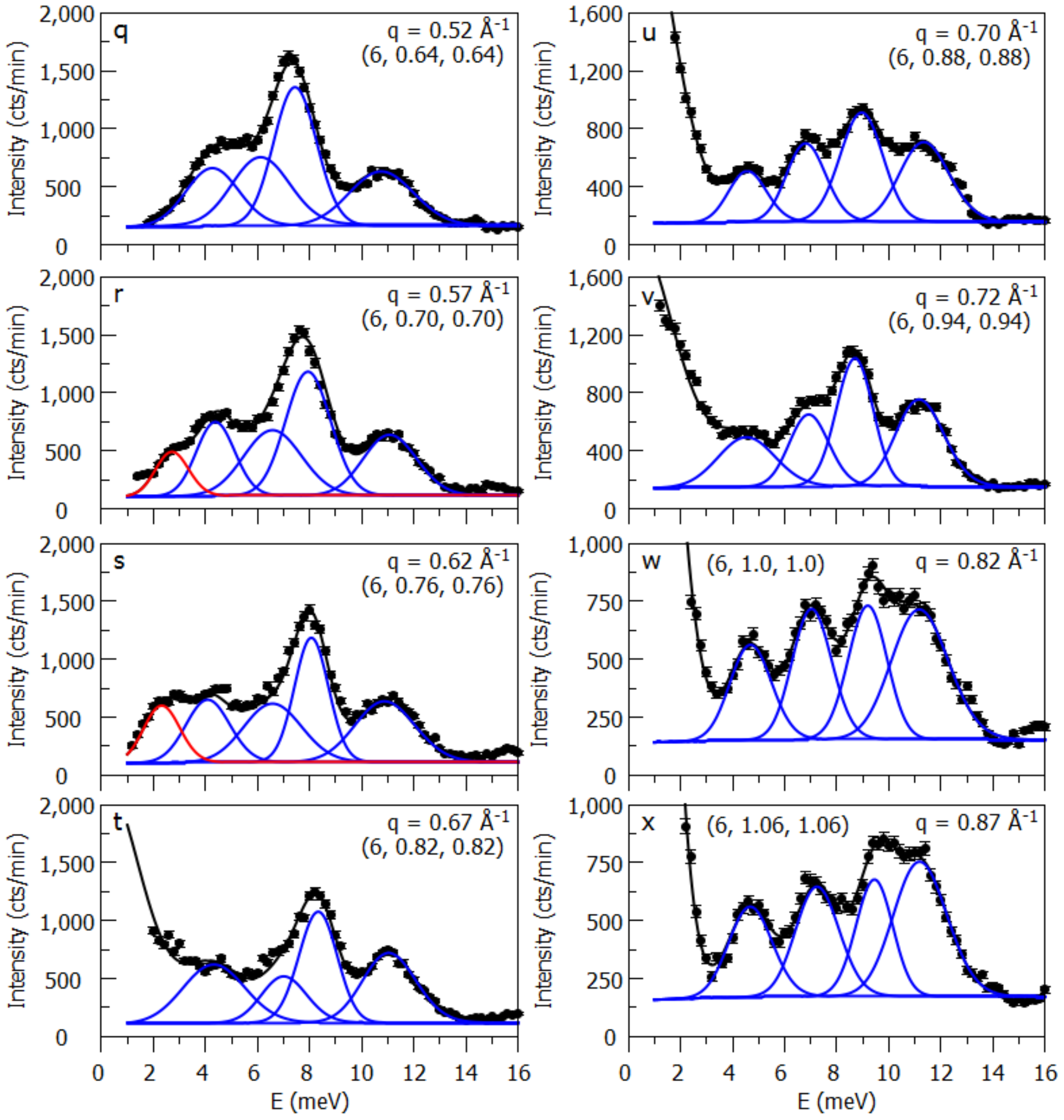
q
q = 0.52 Å-1
(6, 0.64, 0.64)
r
q = 0.57 Å-1
(6, 0.70, 0.70)
s
q = 0.62 Å-1
(6, 0.76, 0.76)
t
q = 0.67 Å-1
(6, 0.82, 0.82)
u
q = 0.70 Å-1
(6, 0.88, 0.88)
v
q = 0.72 Å-1
(6, 0.94, 0.94)
w
(6, 1.0, 1.0)
q = 0.82 Å-1
x
(6, 1.06, 1.06)
q = 0.87 Å-1
Intensity (cts/min)
E (meV)

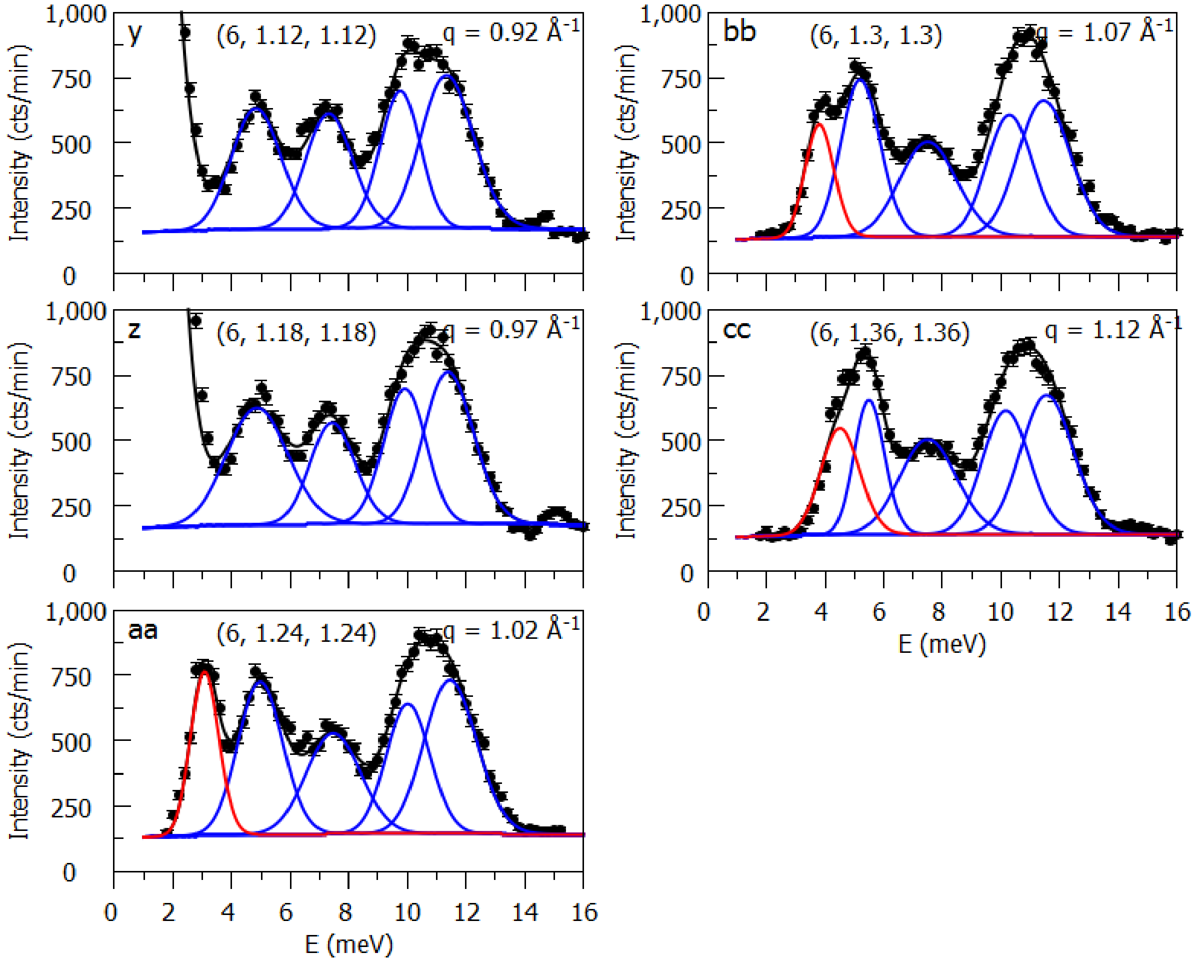

Supplementary Figure 16 : Transverse acoustic (TA) phonon spectra measured in $Ba_{7.81}Ge_{40.67}Au_{5.33}$. *In Panels (a-z, aa-cc) are reported energy scans at constant Q = (600) + q(0kk) throughout the entire Brillouin zone, with k in reciprocal lattice units (rlu). Measurement were performed on the neutron triple axis spectrometer 2T@LLB. The solid black lines represent the global fit of the data (filled circles, vertical error bars are standard deviation from counting statistics) as described in the text. Red lines depict contributions of the TA phonons, blue lines those of the optic-like excitations.*

Supplementary References

1. Hamelin, B. & Bastie, P., Méthode de Laue refocalisée à haute énergie: développements récents, *Le J. Phys. IV,* **8,** Pr5–3, (1998).
2. https://www.ill.eu/instruments-support/instruments-groups/instruments/in22.
3. Rekveldt, M. T., Keller, T. & Golub, R., Larmor precession, a technique for high-sensitivity neutron diffraction, *EPL Europhysics Lett.,* **54,** 342, (2001).
4. Martin, N., Regnault, L.-P. & Klimko, S., Neutron Larmor Diffraction study of the $BaM_2(XO_4)_2$ (M= Co, Ni; X= As, P) compounds, *J. Phys. Conf. Ser.,* **340,** 12012, (2012).
5. Mihalkovič, M. & Widom, M., Ab initio calculations of cohesive energies of Fe-based glass-forming alloys, *Phys. Rev. B,* **70,** 144107, (2004).
6. Johnsen, S., Christensen, M., Thomsen, B., Madsen, G. K. H. & Iversen, B. B., Barium dynamics in noble-metal clathrates, *Phys. Rev. B,* **82,** 184303, (2010).

7. Zhang, H., Borrmann, H., Oeschler, N., Candolfi, C., Schnelle, W., Schmidt, M., Burkhardt, U., Baitinger, M., Zhao, J.-T. & Grin, Y, Atomic Interactions in the p-Type Clathrate I $Ba_8Au_{5.3}Ge_{40.7}$, *Inorganic chemistry*, **50(4)**, 1250–1257, (2011).

8. Ye, Z., Cho, J. Y., Tessema, M. M., Salvador, J. R., Waldo, R. A., Yang, J., Wang, H., Cai, W. Kirkham, M. J., Yang J., Zhang, W., Thermoelectric properties of Au-containing type-I clathrates $Ba_8Au_xGa_{16-3x}Ge_{30+2x}$, *J. Alloys Compd.,* **587,** 747–754, (2014).

9. Chung, J. D., McGaughey, A. J. H. & Kaviany, M., Role of phonon dispersion in lattice thermal conductivity modeling, *J. Heat Transfer,* **126,** 376–380, (2004).

10. http://www-llb.cea.fr/fr-en/pdf/2t1-llb.pdf.

11. Euchner, H., Pailhès, S., Nguyen, L. T. K., Assmus, W., Ritter, F., Haghighirad, A., Grin, Y., Paschen, S. & de Boissieu, M., Phononic filter effect of rattling phonons in the thermoelectric clathrate $Ba_8Ge_{40+x}Ni_{6-x}$, *Phys. Rev. B,* **86,** 224303, (2012).

12. http://www.ill.eu/fr/instruments-support/instruments-groups/instruments/in5. (2015).

13. Ollivier, J. & Mutka, H., IN5 cold neutron time-of-flight spectrometer, prepared to tackle single crystal spectroscopy, *J. Phys. Soc. Japan,* **80,** SB003, (2011).

14. T. Perring, R. A. Ewings & J. V. Duijn, Horace software at http://horace.isis.rl.ac.uk.

15. Mezei, F., Neutron inelastic scattering, *IAEA, Vienna,* 125, (1978).

16. Golub, R. & Gähler, R., A neutron resonance spin echo spectrometer for quasi-elastic and inelastic scattering, *Phys. Lett. A,* **123,** 43–48, (1987).

17. Lu, X., Tam, D. W., Zhang, C., Luo, H., Wang, M., Zhang, R., Leland, W. H., Keller T., Keimer B., Regnault L. P., Thomas A. M. & Pengcheng D., Short-range cluster spin glass near optimal superconductivity in $BaFe_{2-x}Ni_xAs_2$, *Phys. Rev. B,* **90,** 24509, (2014).

18. Klaus Habicht. SERESCAL.

19. Computational Materials Physics, V. http://www.vasp.at/.

20. K., P. http://wolf.ifj.edu.pl/phonon/Public/phrefer.html. Software phonon, version 4.28. (2005).

21. Pailhes, S., Euchner, H., Giordano, V. M., Debord, R., Assy, A., Gomes, S., Bosak, A., Machon, D, Paschen, S. & De Boissieu M., Localization of Propagative Phonons in a Perfectly Crystalline Solid, *Phys. Rev. Lett.,* **113,** 25506, (2014).

22. T. Tadano Alamode. http://sourceforge.net/projects/alamode/.

23. Slack, G. A., Thermal conductivity of elements with complex lattices: B, P, S., *Phys. Rev.,* **139,** A507, (1965).

24. Roufosse, M. & Klemens, P. G. Thermal conductivity of complex dielectric crystals. *Phys. Rev. B* **7,** 5379 (1973).

25. de Boissieu, M., Currat, R., Francoual, S. & Kats, E., Sound-mode broadening in quasicrystals: A simple phenomenological model, *Phys. Rev. B,* **69,** 54205, (2004).

26. Janssen, T., Chapuis, G. & De Boissieu, M., *Aperiodic crystals: from modulated phases to quasicrystals*, (Oxford: Oxford University Press, 2007, 2007).

27. de Boissieu, M., Francoual S., Mihalkovic, M., Shibata, K., Baron, A. F. Q., Sidis, Y. Ishimasa, T., Wu, D., Lograsso, T., Regnault, L-P., Gähler, F., Tsutsui, S., Hennion, B., Bastie, P., Sato, T. J., Takakura, H., Currat, R. & Tsai, A-P., Lattice dynamics of the Zn-Mg-Sc icosahedral quasicrystal and its Zn--Sc periodic 1/1 approximant, *Nat. Mater.,* **6,** 977–984, (2007).
